# Novel myostatin-specific antibody enhances muscle strength in muscle disease models

**DOI:** 10.1038/s41598-021-81669-8

**Published:** 2021-01-25

**Authors:** Hiroyasu Muramatsu, Taichi Kuramochi, Hitoshi Katada, Atsunori Ueyama, Yoshinao Ruike, Ken Ohmine, Meiri Shida-Kawazoe, Rie Miyano-Nishizawa, Yuichiro Shimizu, Momoko Okuda, Yuji Hori, Madoka Hayashi, Kenta Haraya, Nobuhiro Ban, Tatsuya Nonaka, Masaki Honda, Hidetomo Kitamura, Kunihiro Hattori, Takehisa Kitazawa, Tomoyuki Igawa, Yoshiki Kawabe, Junichi Nezu

**Affiliations:** 1 Research Division, Chugai Pharmaceutical Co., Ltd., Tokyo, 103-8324 Japan; 2 Chugai Pharmabody Research Pte. Ltd., 3 Biopolis Drive, #07-11 to 16, Synapse, Singapore, 138623 Singapore

**Keywords:** Protein design, Antibody therapy

## Abstract

Myostatin, a member of the transforming growth factor-β superfamily, is an attractive target for muscle disease therapy because of its role as a negative regulator of muscle growth and strength. Here, we describe a novel antibody therapeutic approach that maximizes the potential of myostatin-targeted therapy. We generated an antibody, GYM329, that specifically binds the latent form of myostatin and inhibits its activation. Additionally, via “sweeping antibody technology”, GYM329 reduces or “sweeps” myostatin in the muscle and plasma. Compared with conventional anti-myostatin agents, GYM329 and its surrogate antibody exhibit superior muscle strength-improvement effects in three different mouse disease models. We also demonstrate that the superior efficacy of GYM329 is due to its myostatin specificity and sweeping capability. Furthermore, we show that a GYM329 surrogate increases muscle mass in normal cynomolgus monkeys without any obvious toxicity. Our findings indicate the potential of GYM329 to improve muscle strength in patients with muscular disorders.

## Introduction

Myostatin, also known as growth differentiation factor 8 or GDF8, is a member of the transforming growth factor (TGF)-β superfamily^1^. Genetic loss of myostatin is known to cause hypermuscular phenotypes in animals including hyperplasia and hypertrophy of skeletal muscle fiber in mice^1–3^; hypertrophy of muscle fiber in cattle^4–6^; and improved physical function in dogs^7^. In addition, a human case of homozygous loss-of-function mutation of the myostatin gene was reportedly associated with increased muscle mass and strength^8^. Myostatin is predominantly expressed in skeletal muscle and synthetized as a precursor called pro-myostatin that is cleaved by a furin to give the latent myostatin/latent complex, which will be cleaved by proteases such as bone morphogenetic protein 1 (BMP1) or Tolloid-like protein 2 (TLL2) allowing the release of the mature/active dimer^1,9–13^. The mature form of myostatin binds and activates cognate receptors including ALK4/5 (type I receptor) and ActRIIA/B (type II receptor) on the surface of muscle cells; this activation results in the inhibition of protein synthesis and enhancement of protein degradation, thus leading to muscular atrophy^14^. Myostatin is now widely accepted as the key negative regulator of skeletal muscle growth and strength.

Pharmacological intervention to inhibit the myostatin pathway is therefore considered an attractive therapeutic approach for various types of muscle disorders, such as muscular dystrophy and atrophy, for which no effective treatment is currently available. Multiple therapeutic agents targeting the myostatin pathway have been and are being tested in clinical studies^15^. These include the anti-mature myostatin antibodies LY2495655/landogrozumab^16,17^ and PF-06252616/domagrozumab^18,19^; an anti-mature myostatin adnectin (BMS-986089)^20^; a soluble ActRIIb-IgG fusion protein (ACE-031/ramatercept)^21^; and a modified follistatin-IgG fusion protein (ACE-083)^22^. Although some biological responses have been observed in early clinical studies, the clinical outcomes were not satisfactory, especially in terms of improving muscle function^17,21,23^. Therefore, a new therapeutic approach with better efficacy is needed.

The aforementioned agents in clinical studies inhibit not only myostatin but also other TGF-β superfamily members, such as GDF11, which has a high sequence similarity with myostatin^18,24,25^. However, the role of GDF11 in muscle growth and strength is poorly understood, and whether the inhibition of GDF11 is beneficial for the treatment of muscle diseases is unclear^26–30^. We therefore attempted to generate an antibody that specifically blocks myostatin. Since the mature domains of myostatin and GDF11 have 90% sequence similarity, myostatin-specific neutralizing antibodies that bind this domain are difficult to generate. Thus, we tried to generate antibodies that specifically prevent myostatin activation to the mature form by binding the prodomain of the latent form of myostatin, which has a lower sequence similarity (52%) with the prodomain of GDF11^31^.

We also hypothesize that the neutralization of myostatin in the muscle tissue microenvironment by the current anti-myostatin agents is insufficient. Muscle fibers are reported to contain high levels of the precursor form of myostatin^13^; the amount of antibodies around the muscle fibers might not be sufficient to completely neutralize the mature myostatin generated from the precursor molecules of myostatin due to poor antibody penetration in the muscles^32,33^. To overcome this hurdle, we added a “sweeping function” to the antibody based on the novel antibody engineering technology (“sweeping antibody technology”) that we have recently developed^34–36^. This technology incorporates two core elements into the “sweeping antibody”: (1) a fragment crystallizable (Fc) domain with enhanced affinity to the FcγRIIb receptor^35^, and (2) an antigen-binding fragment (Fab) domain that allows pH-dependent binding of the antibody to its antigen^34,37^. We have previously reported the generation and characterization of a “sweeping antibody” and have described its pharmacokinetic properties^36^. The antibody and its antigen first form an immune complex; this complex is captured by FcγRIIb on the surface of certain types of endothelial and immune cells and is then internalized^38,39^. FcγRIIb-mediated internalization, particularly in liver sinusoidal endothelial cells (LSEC) where FcγRIIb is predominantly expressed, is considered a major physiological pathway for systemic immune complex clearance^39^. The sweeping antibody takes advantage of this mechanism through the engineered Fc region that has high affinity to FcγRIIb. The acidic pH (~ pH 5.8) in the endosome then triggers the dissociation of the internalized immune complex, and only the free antibody is carried back to the cell surface by the neonatal Fc receptor (FcRn), while the free antigen is shuttled to the lysosome for degradation^36,37^. FcRn is an Fc receptor located on the endosomal membrane and the cell surface, and it is responsible for recycling IgGs and albumin taken up spontaneously from the endosome^40^ to maintain their physiological concentrations in the plasma. By repeating this “capture-and-release cycle,” the engineered antibody can reduce the amount of antigen outside the cell, as if it is “sweeping” it.

In this study, we report the generation and in vivo characterization of an antibody we named GYM329, which has myostatin-specific blockade and sweeping capabilities. Specifically, we analyzed the effects of GYM329 and its functional equivalent in mouse models of muscle disease. We further demonstrated the effects of GYM329 and its surrogate in cynomolgus macaques. We demonstrate that this antibody exhibits better muscle strength improvement activity than conventional anti-myostatin agents, showing that GYM329 is a potent novel agent for the treatment of muscle diseases.

## Results

### Generation and characterization of GYM329, latent myostatin-specific antibody with pH-dependent binding properties

Anti-latent myostatin antibodies were generated in rabbits by alternatively immunizing the animals with recombinant human and mouse latent myostatin to enrich cross-reactive clones. Screening of B cell supernatants from the immunized rabbits through a binding assay identified clones that specifically bind the latent and not the mature myostatin. They were then functionally screened through the Smad3/4-binding elements-driven secreted alkaline phosphatase (SEAP) reporter gene assay. We assessed the candidate antibodies’ inhibitory activity against BMP1-mediated activation of myostatin, and MST1032 was finally selected as the lead antibody based on its strong activity.

**Figure 1:**
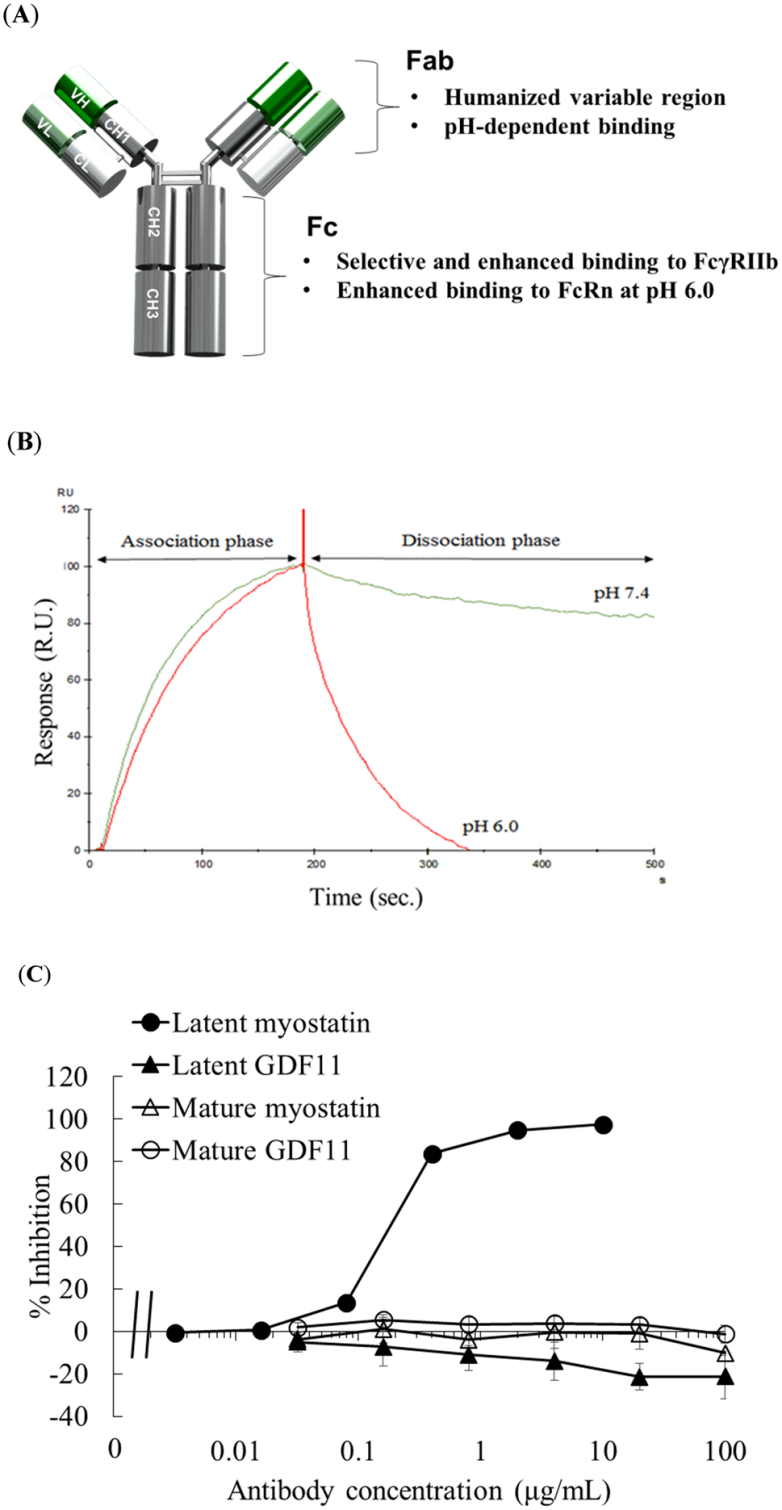
Generation of the anti-latent myostatin sweeping antibody, GYM329. (**A**) Summary of the characteristics engineered into GYM329. (**B**) Surface plasmon resonance analysis illustrating pH-dependent binding of GYM329 to latent myostatin. Binding of GYM329 to human latent myostatin was monitored at pH 7.4 in the association phase (0 to 180 s) and dissociation of GYM329 from latent myostatin at either pH 6.0 or 7.4 were assessed in the dissociation phase (from 180 s). The sensorgram was normalized by adjusting the latent myostatin binding response to ‘100.’ RU: resonance unit. (**C**) Inhibitory effects of GYM329 on latent myostatin, latent GDF11, mature myostatin, and mature GDF11. Latent myostatin or latent GDF11: 3 nmol/L; mature GDF11 or mature myostatin: 5 ng/mL. Mean ± SD (n = 3).

The variable domain of MST1032 was humanized and engineered to confer pH-dependent binding essential for the sweeping function through a comprehensive mutagenesis method described elsewhere^41^. For the Fc region, human IgG1 was chosen as template and was engineered for selective and enhanced binding to the human FcγRIIb and for stronger affinity to FcRn in acidic pH conditions. The resulting antibody was named GYM329 (Fig. 1A) and was subjected to subsequent characterization.

The representative results of the surface plasmon resonance (SPR) analysis demonstrating the pH-dependent binding of GYM329 to the human latent myostatin are shown in Fig. 1B. A higher dissociation rate was observed at pH 6.0 than at pH 7.4 in the dissociation phase of the analysis. The *k*_a_, *k*_d_, and *K*_D_ values at pH 7.4 for the human, cynomolgus monkey, and mouse latent myostatins were nearly equal (Table S1). To assess the binding affinity of GYM329 Fc to various FcγR subtypes, GYM329 and a reference wild type human IgG1 (with trastuzumab Fab) were captured onto an SPR chip. The amount of soluble human FcγR recombinant proteins bound to the antibodies were compared. The ratios (GYM329-bound FcγR divided by the reference IgG1-bound FcγR) are shown in Table S2. FcγRIIb had higher affinity to GYM329 than to IgG1 (> 5-folds), and other FcγRs had much lower affinities to GYM329. This data demonstrates that the Fc of GYM329 has enhanced selectivity and affinity to FcγRIIb. The binding affinity of GYM329 and the reference IgG1 to the human and cynomolgus monkey FcRn at pH 6.0 were determined by SPR analysis (Table S3). GYM329 had stronger affinity to FcRn than IgG1 in acidic conditions. This is indicative of a long serum half-life for GYM329 as there is more efficient FcRn-mediated recycling of the internalized antibody from the endosome, as observed in other engineered antibodies with the same property.

The effects of different GYM329 doses on the inhibition of BMP1-facilitated and spontaneous activation of human, cynomolgus monkey, and mouse latent myostatins were assessed using the SEAP reporter gene assay. The half-maximal inhibitory concentrations (IC_50_) were comparable between the species (Table S4). We also confirmed that the inhibitory effect of GYM329 is specific to latent myostatin, as no inhibition of mature myostatin or latent/mature GDF11 was observed (Fig. 1C).

### GYM329-induced muscle mass increase and muscle strength enhancement in three different mouse models of muscle disease

To determine whether GYM329 could increase muscle mass and enhance muscle strength, we tested three different mouse models of muscle disease. To minimize the production of mouse antibodies against human IgG1-derived constant regions of GYM329, we generated its murine functional equivalent, GYM-mFc, by fusing mouse IgG1-derived constant regions to the Fab domain of GYM329. GYM-mFc was confirmed to have identical inhibitory potency against activation of latent myostatin as GYM329 in vitro (Fig. S1).

**Figure 2:**
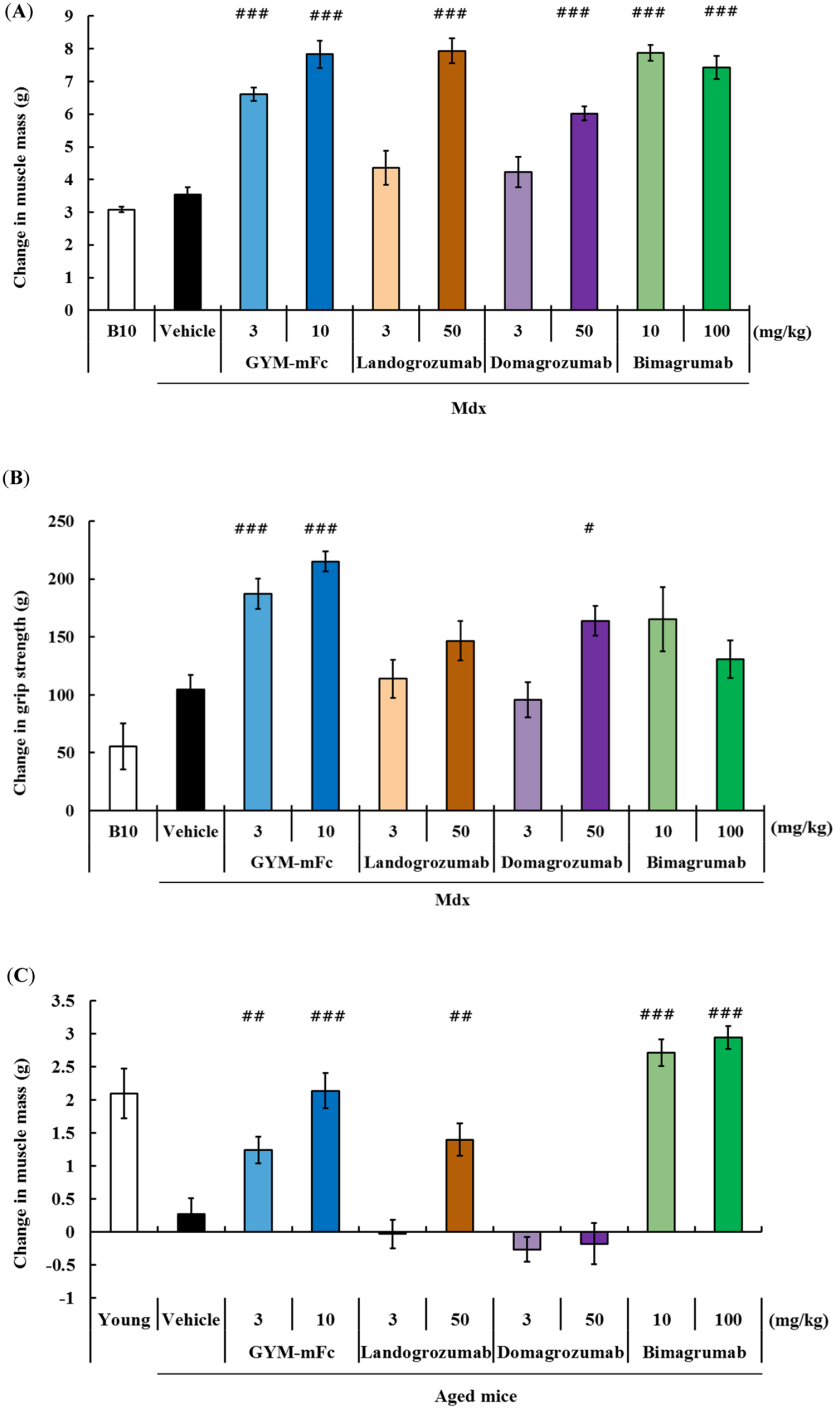

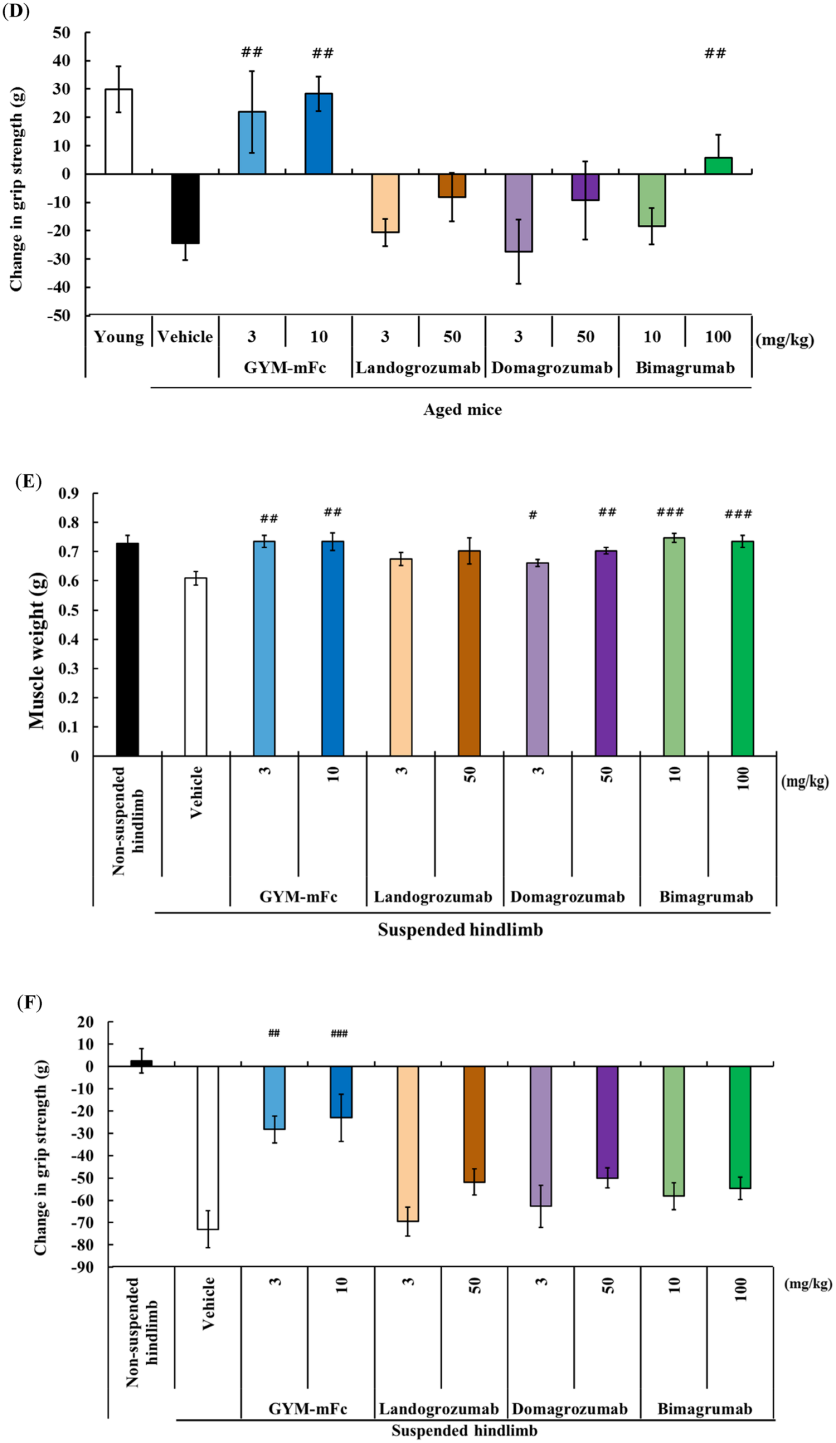
The mouse surrogate of GYM329 (GYM-mFc) strongly increases muscle strength and muscle mass in three mouse models of muscle disease. Changes in (**A**) whole-body muscle mass and (**B**) appendicular grip strength 4 weeks after antibody injection in *mdx* mice (n = 5–6 per group). Changes in (**C**) whole-body muscle mass and (**D**) appendicular grip strength 4 weeks after antibody injection in aged mice (n = 9–10 per group). (**E**) Hindlimb muscle weights after 2 weeks of hindlimb suspension in the muscular atrophy model. Hindlimb muscles (quadriceps, gastrocnemius, tibialis anterior, EDL, and soleus) were collected and weighed 2 weeks after treatment. (**F**) Changes in hindlimb grip strength after pre-treatment with the antibodies in the muscular atrophy model. Data represent the changes in hindlimb grip strength during 2 weeks of the hindlimb suspension period. Data represent mean ± SEM (n = 5–6 per group). ^#^*P* < 0.025, ^##^*P* < 0.005, and ^###^*P* < 0.0005, Williams’ test compared with the vehicle (150 mmol/L NaCl, 20 mmol/L L-Histidine, pH 6.0) group.

We first evaluated the activity of GYM-mFc in a Duchenne muscular dystrophy (DMD) mouse model, C57BL/10-*mdx* Jic (*mdx* mice). We compared the activity of GYM-mFc with the activity of three different clinically tested anti-myostatin blocking antibodies that we generated in-house based on the sequence information in International Immunogenetics Information System (IMGT) database (http://www.imgt.org). These include two mature myostatin-neutralizing antibodies based on the sequences of landogrozumab and domagrozumab and the anti-activin receptor II antibody bimagrumab. Two doses (high and low) of all the agents were evaluated. Almost similar maximum effects on muscle mass increment were observed with the high doses of all tested agents (Fig. 2A). On the other hand, significant grip strength enhancement was observed in mice treated with GYM-mFc (at both doses) and with domagrozumab (at the high dose) but not in mice treated with the other agents (Fig. 2B).

We evaluated the activity of these agents in aged mice (seventy-nine weeks old), which exhibit sarcopenic characteristics, such as decreased muscle mass per bodyweight, impaired muscle strength, and low physical activity (Fig. S2). An increase in muscle mass up to levels comparable to those found in young mice was observed only in the aged mice treated with GYM-mFc or with bimagrumab (Fig. 2C). At a high dose of landogrozumab (50 mg/kg), a small increase in muscle mass was observed, but no effect was observed in treatment with the lower dose of landogrozumab or with any dose of domagrozumab. Intriguingly, a remarkable enhancement in grip strength was observed only in GYM-mFc-treated mice, reaching levels that are comparable to those in young mice even at a lower treatment dose (3 mg/kg, Fig. 2D). Much weaker grip strength enhancement was seen at the high bimagrumab dose (100 mg/kg), and no enhancement was observed in mice treated with landogrozumab and domagrozumab.

Lastly, the activity of these agents was assessed in a muscular atrophy model, which was recreated by suspending the hindlimb of the mice for two weeks. Muscle tissue weight reduction was seen in the vehicle-treated muscular atrophy group compared to the non-suspension control group, which was completely suppressed by treatment with GYM-mFc, domagrozumab, and bimagrumab, but not by landogrozumab (Fig. 2E). Remarkable impairment in grip strength was also induced by hindlimb suspension, and it was significantly ameliorated only by GYM-mFc treatment (Fig. 2F).

In all these studies, GYM-mFc had the most potent effects on muscle mass and muscle strength of all the tested anti-myostatin agents.

### Negative contribution of GDF11 signaling blockade to muscle strength enhancement

We next aimed to determine the possible molecular mechanism underlying the superior effects of the GYM329 surrogate over the other agents, particularly for muscle strength enhancement, which is more important therapeutically than muscle mass increment. Two fundamental differences exist between GYM329 and the other agents: specificity to myostatin and the sweeping function. GYM329 can specifically inhibit myostatin signaling by binding latent myostatin and suppressing its activation, whereas landogrozumab and domagrozumab bind mature myostatin and GDF11 with similar affinity to both molecules, thereby equally inhibiting both (Fig. S3). Bimagrumab is an antibody against ActRII that blocks its interaction with multiple TGF-β superfamily ligands including myostatin, GDF11, and activin^28,42^. In addition, the mechanism of action of all these conventional antibodies includes simple neutralization, while GYM329 has a sweeping function that reduces antigen levels by forcing internalization via FcγRs.

**Figure 3:**
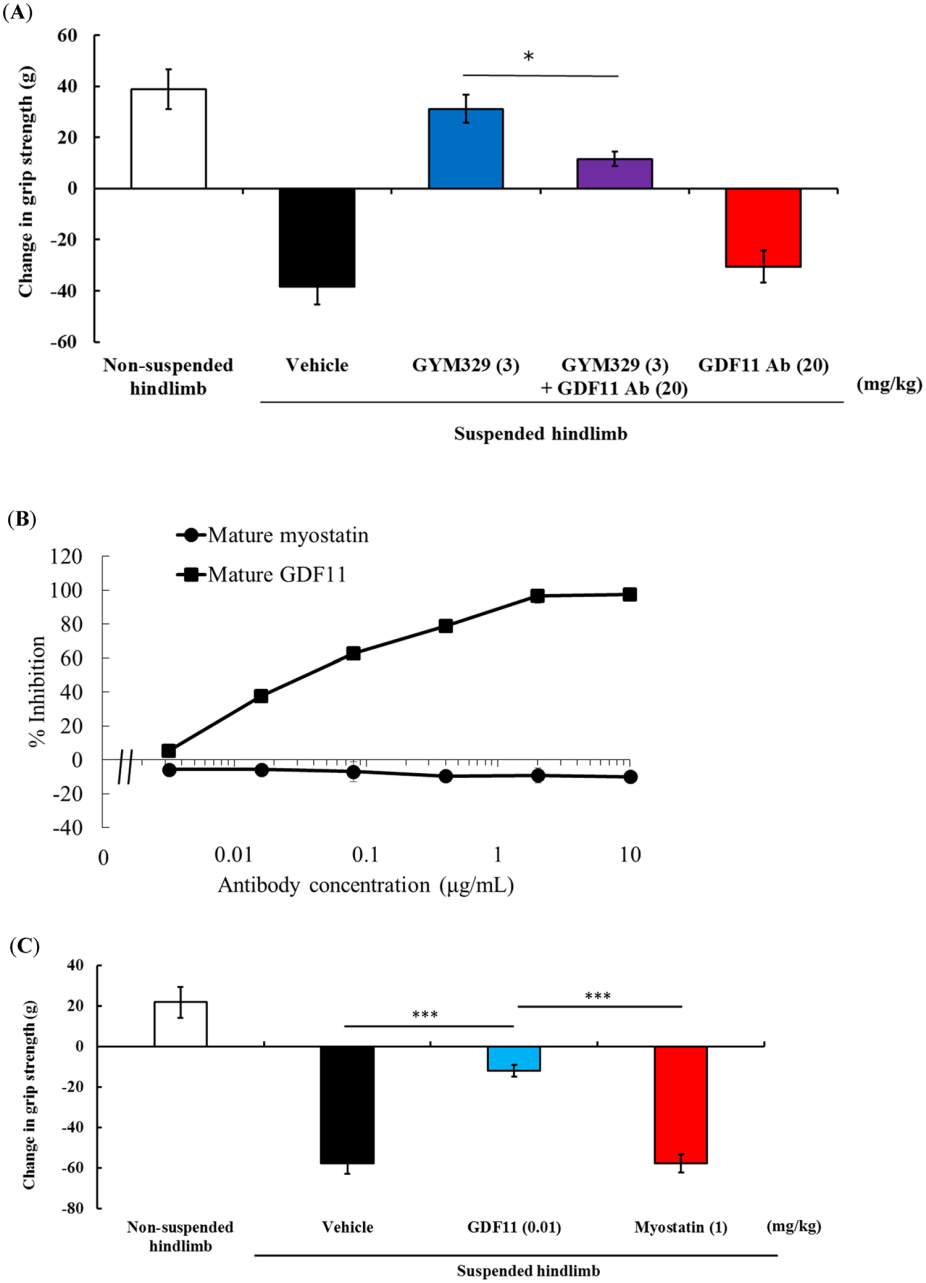
GDF11 signaling blockade negatively affects muscle strength in the muscular atrophy model. (**A**) Changes in hindlimb grip strength 2 weeks after treatment (n = 5–6 per group). Antibodies were administered (i.v.), followed by hindlimb suspension on day 0. Two weeks after hindlimb suspension, hindlimb muscles (quadriceps, TA, EDL, soleus, and gastrocnemius) were isolated and weighed (n = 5–6 per group). Data represent mean ± SEM. *P < 0.05 with Student’s *t*-test. (**B**) Inhibitory activities of antibodies against mature GDF11 or mature myostatin; 5 ng/mL of mature GDF11 (square) or mature myostatin (circle) was used for the reporter gene assay. Data are presented as mean ± SD (n = 3). (**C**) Changes in hindlimb grip strength over three days. (n = 6 per group). Recombinant GDF11 or myostatin were administered (i.p.) on day 0, 1, and 2. Data represent mean ± SEM (n = 6 per group). ***P < 0.001 with Tukey test performed without the non-suspension group.

We first determined whether the specificity of GYM329 to myostatin is involved in its superior efficacy in muscle strength enhancement. This was investigated in a hindlimb suspension muscular atrophy model because direct testing of GYM329 with the human IgG sequence is possible in this model with the use of mice with severe combined immunodeficiency (SCID mice). As observed with the surrogate antibody (Fig. 2F), GYM329 (3 mg/kg) significantly enhanced grip strength in the hindlimb suspension mouse model, reaching levels comparable to those in the non-suspended control mice (Fig. 3A). To assess the influence of the GDF11 signaling blockade in this phenomenon, we generated a neutralizing antibody specific to mature GDF11. We have confirmed that this antibody binds mature GDF11 but not myostatin (Fig. S4), and that it specifically neutralizes GDF11-mediated signaling (Fig. 3B). Interestingly, we found that the muscle strength enhancement induced by GYM329 was significantly suppressed by combined treatment with the anti-GDF11 antibody (Fig. 3A). Meanwhile, anti-GDF11 antibody treatment alone did not show any muscle strength enhancement activity.

We next examined the effect of GDF11 itself on muscle strength enhancement. Administration of recombinant mature GDF11 (0.01 mg/kg bodyweight, three times, intraperitoneal) significantly suppressed the reduction of muscle strength induced by hindlimb suspension (Fig. 3C). On the other hand, recombinant myostatin, which was confirmed to have similar potency as the recombinant mature GDF11 protein in the reporter gene assay described above (Fig. S5), did not have any suppressive effects on muscle strength reduction. Taken together, these data suggest that GDF11 and myostatin act in opposing directions in terms of muscle strength enhancement, where inhibition of GDF11 signaling negatively impacts muscle strength enhancement.

### Contribution of the sweeping function of GYM329 to its muscle strength enhancement activity

**Figure 4:**
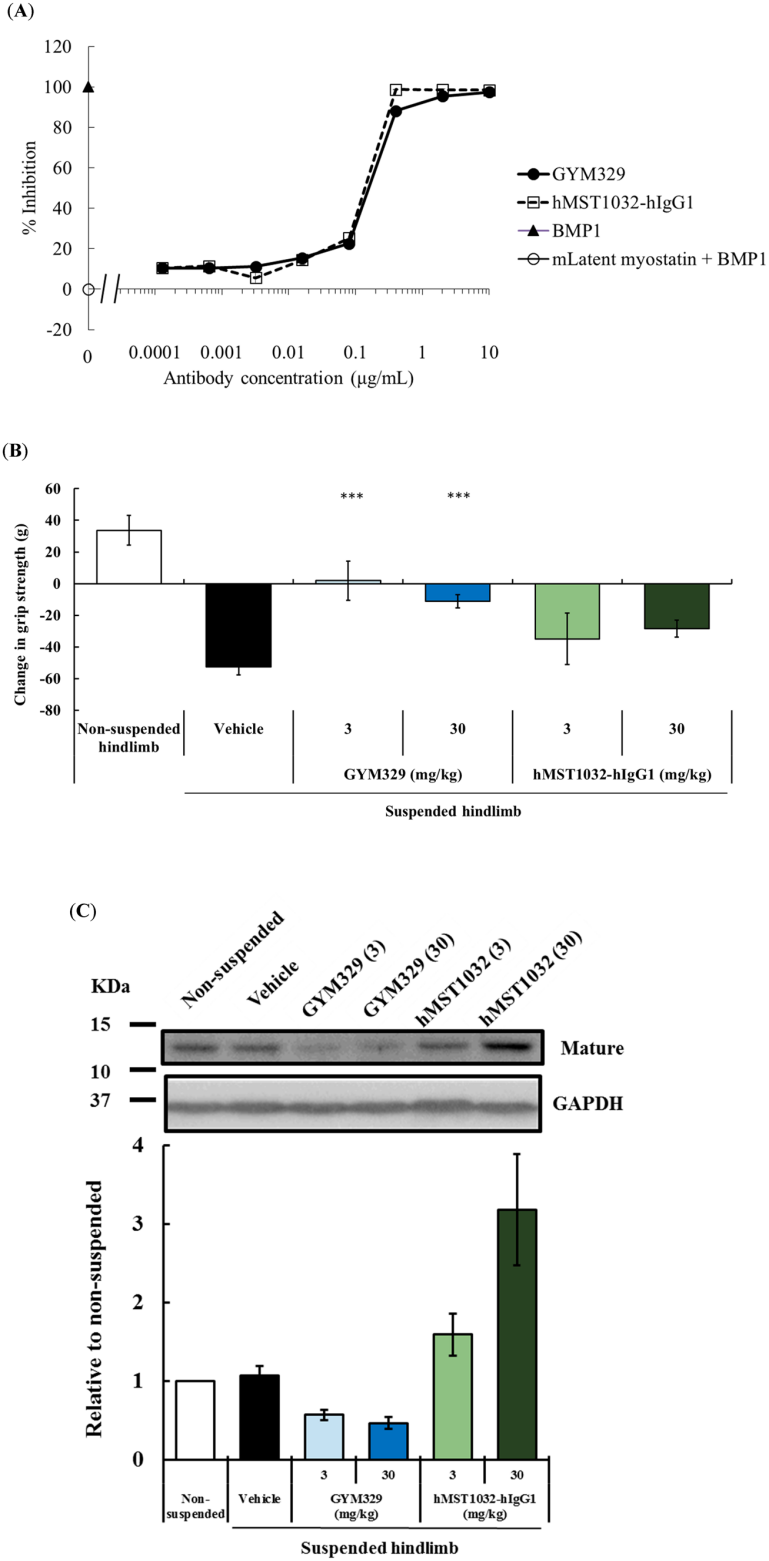

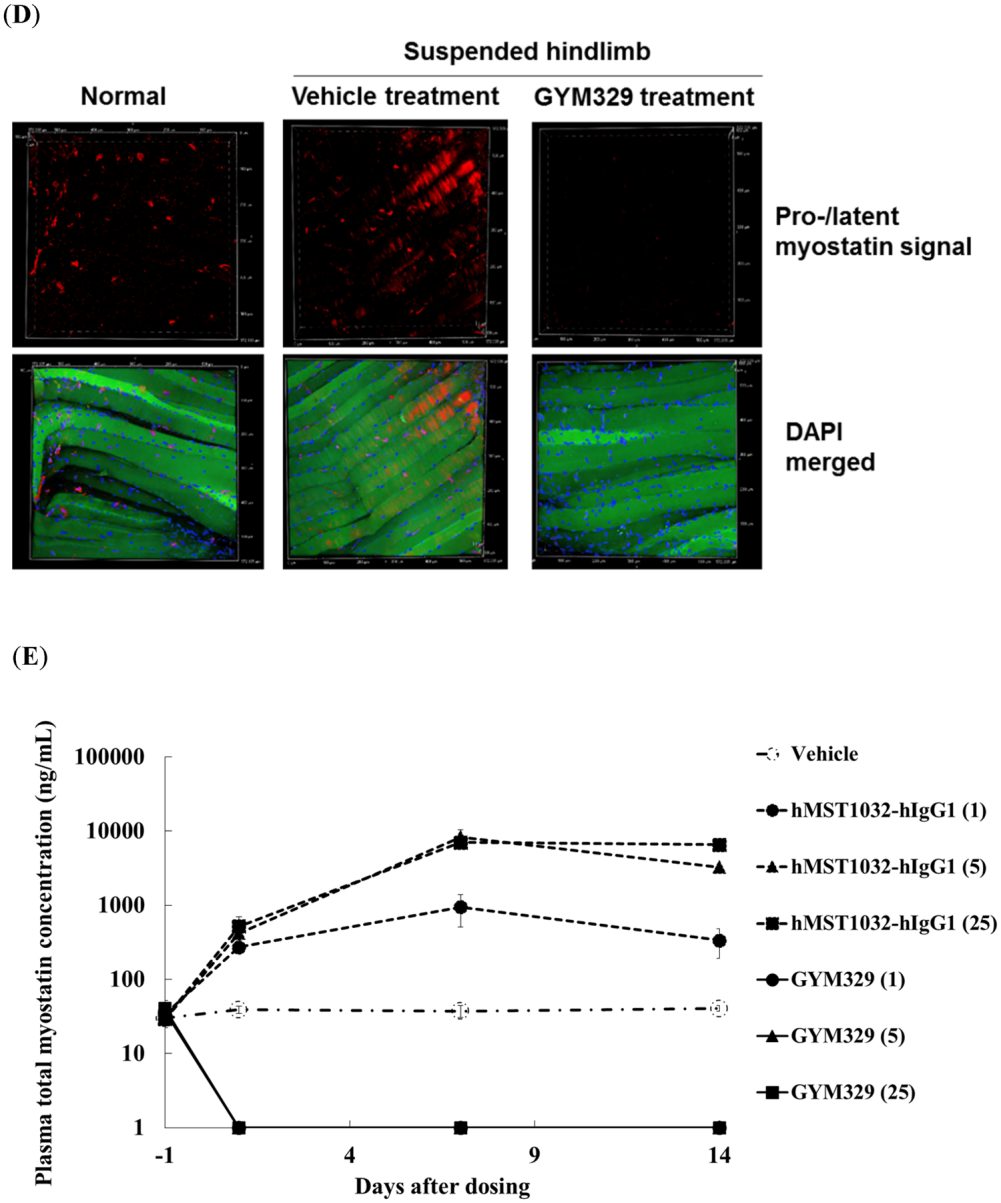
The sweeping function of GYM329 positively contributes to muscle strength improvement in the muscular atrophy model. (**A**) Inhibition of BMP1-mediated activation of mouse latent myostatin (3 nmol/L) by the anti-latent myostatin sweeping antibody, GYM329, and the non-sweeping antibody, hMST1032-hIgG1, determined by the Smad reporter gene assay. Data represent mean ± SD (n = 3). (**B**) Changes in hindlimb grip strength one week after injection (i.v.) with GYM329 or hMST1032-hIgG1. ***P < 0.0005 using Williams’ test compared with the vehicle group. (**C**) Mature myostatin levels in isolated mouse quadriceps after the in vivo study in (**B**). A representative western blotting image and quantified results are shown (the non-suspension group was assigned the value of 1; n = 6; mean ± SEM). Full length blots are presented in Supplementary Information (Fig. S7). (**D**) Confocal imaging of whole mounts of the extensor digitorum longus (EDL) in the muscular atrophy model. Pro-/latent myostatin localized in the extracellular space of the skeletal muscles was labeled by the anti-pro-myostatin/latent myostatin antibody, MST1098-rabbit IgG, followed by the secondary anti-rabbit-IgG Alexa Fluor 568 (red). Representative confocal images of muscles from the non-suspension (left) and hindlimb suspension group (vehicle treatment, middle; GYM329 treatment, right) are shown. (**E**) Plasma concentration–time curve of total myostatin in normal mice after administration of GYM329 or hMST1032-hIgG1. GYM329 or hMST1032-hIgG1 was intravenously injected into normal mice on day 0. Total plasma myostatin concentration was measured by the electrochemiluminescence immunoassay (n = 6, mean ± SD). Total myostatin is the C-terminal domain of myostatin including both antibody-bound and unbound, or other protein-bound myostatin forms. Values < 1 ng/mL were considered to be below the limit of quantification (BLQ).

We then explored the possible contribution of the sweeping function of GYM329 to its superior muscle strength enhancing activity over other anti-myostatin agents. We generated a reference antibody, hMST1032-hIgG1, which has a humanized MST1032 Fab without the pH-dependent antigen binding property, and a wild-type human IgG1 Fc. The Smad reporter gene assay showed that hMST1032-hIgG1 has the same potency as GYM329 in inhibiting the BMP1-mediated activation of latent myostatin (Fig. 4A). Due to the lower affinity of the wild-type human IgG1 to FcγRs, particularly to FcγRIIb, compared to the engineered GYM329 Fc, the immune complex formed by hMST1032-hIgG1 and latent myostatin was thought to be captured and internalized into the cell less efficiently compared to the GYM329 and latent myostatin combination. Furthermore, due to the lack of pH-dependent binding in hMST1032-hIgG1, latent myostatin is not expected to dissociate from the antibody in the acidic endosome, and the latent myostatin bound to the antibody is brought back to the extracellular space. This would result in a lower clearance of latent myostatin, even if it is taken up into the cell via a less efficient internalization of the immune complex. In the absence of the two properties essential for the sweeping function, hMST1032-hIgG1 serves as a non-sweeping reference for GYM329 with the same potency in the myostatin functional blockade.

Muscle strength enhancement by GYM329 and hMST1032-hIgG1 was compared in the hindlimb suspension muscular atrophy model (Fig. 4B). We found that hMST1032-hIgG1 treatment led to lower muscle strength enhancement even when administered at a high dose (30 mg/kg), suggesting that the sweeping function of GYM329 contributes to its potent capacity to enhance muscle strength. A pharmacokinetic study of GYM329 and hMST1032-hIgG1 showed that hMST1032-hIgG1 has a slightly longer plasma half-life than GYM329 (Fig. S6); therefore, the superior activity of GYM329 in this experiment is not due to differences in antibody exposure.

Western blot analysis for mature myostatin in quadriceps collected from the animals in the same experiment revealed a reduction in the levels of mature myostatin by GYM329 treatment but not by hMST1032-hIgG1 treatment (Fig. 4C, Fig. S7). This was further confirmed by fluorescent immunostaining (Fig. 4D) that detects both pro-myostatin and latent myostatin. The signals appeared to be stronger in the *extensor digitorum longus* (EDL) muscle of the hindlimb that had been suspended to induce muscular atrophy (vehicle treatment, middle) compared to non-manipulated animals (left). The signal was greatly suppressed in the muscles treated with GYM329 (right), suggesting that myostatin sweeping occurred in the muscle. GYM329 treatment reduced the baseline plasma myostatin levels (mature + latent myostatin; Fig. 4E), which is another indication of the sweeping function of GYM329. Treatment with the non-sweeping variant hMST1032-hIgG1 enhanced the myostatin staining signal in the muscle and increased the level of total myostatin in the plasma, which indicate lower muscle and systemic clearance of myostatin. The sweeping effect exhibited by GYM-mFc was similar to the effects of GYM329 (Fig. S8). Taken together, the sweeping function of GYM329 and its myostatin specificity contributed to its superior activity.

### Muscle mass increase and plasma myostatin sweeping activity of the GYM329 surrogate in cynomolgus monkeys

Finally, we investigated the activity of GYM329 in cynomolgus monkeys using a functional equivalent, GYM-cyFc. Although the affinity of the GYM329 Fc to human FcγRIIb was higher than that of the wild-type human IgG1 (Table S2), its affinity to cynomolgus FcγRIIa and cynomolgus FcγRIIb was similar (Table S5). We therefore generated another engineered Fc with enhanced affinity to cynomolgus FcγRIIa and FcγRIIb from the Fc of GYM329, which is combined with the same Fab of GYM329 resulting in GYM-cyFc. We have confirmed that GYM-cyFc selectively binds cynomolgus monkey FcγRIIa and FcγgRIIb (Table S5), with lower binding activity to FcγRI and FcγRIII. The IC_50_ of GYM-cyFc against BMP1-mediated activation of cynomolgus myostatin in vitro was determined to be 0.199 μg/mL, which is comparable to the IC_50_ of GYM329 against the activation of human latent myostatin (0.182 μg/mL). These data suggest that GYM-cyFc is a functional equivalent of GYM329 that may be used for studies in cynomolgus monkeys.

**Figure 5:**
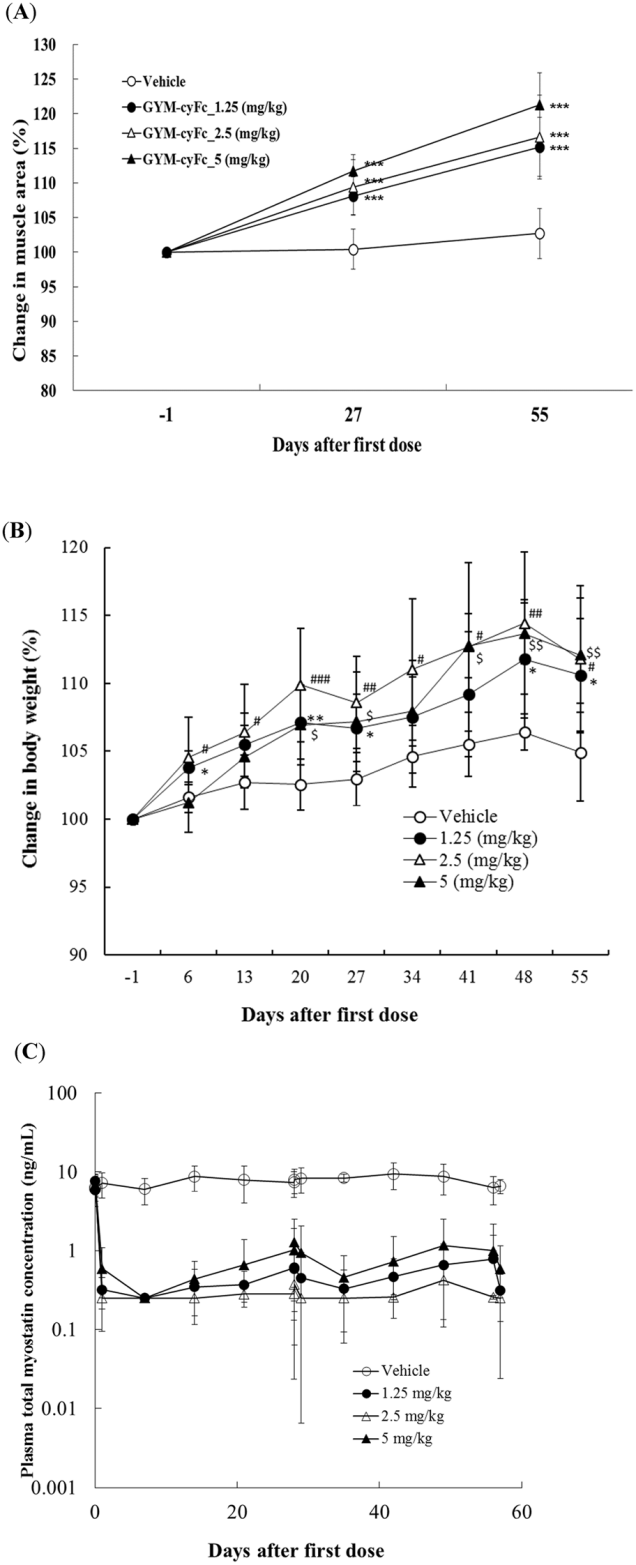
The monkey GYM329 surrogate antibody, GYM-cyFc, increases muscle area and bodyweight, and reduces total myostatin levels in cynomolgus monkeys. (**A**) Muscle section area determined by MRI are presented as the sum of the area of the quadriceps femoris, brachialis, and elector spinae on days − 1 (Pre), 27 (4 weeks), and 55 (8 weeks) from the administration (i.v.) of the first dose of GYM-cyFc. GYM-cyFc was administered every 4 weeks for 2 months. From the results of anti-drug antibody (ADA) and pharmacokinetic analyses, ADA-positive animals were excluded (vehicle group: n = 6; 1.25 mg/kg dose: n = 9; 2.5 mg/kg dose: n = 6; and 5 mg/kg dose: n = 5). Data represent mean ± SD. ***P < 0.0005, Williams’ test for multiple comparisons to the vehicle group. (**B**) The rates of increase in bodyweight relative to baseline after the 1^st^ administration of GYM-cyFc into cynomolgus monkeys. Data represent mean ± SD. *^/#/$^*P* < 0.025, **^/##/$$^*P* < 0.005, ***^/###/$$$^*P* < 0.0005, Williams test for multiple comparisons to the vehicle group (*: 1.25 mg/kg; ^#^: 2.5 mg/kg; and ^$^: 5 mg/kg). (**C**) Plasma concentration–time curve of total myostatin. Total myostatin is the C-terminal domain of myostatin including both antibody-bound and unbound, or other protein-bound myostatin forms. Values under 0.25 ng/mL were considered to be below the limit of quantification (BLQ). Data represent mean ± SD.

Administration (intravenous, i.v.) of 1.25, 2.5, or 5 mg/kg GYM-cyFc to female cynomolgus monkeys (n = 10 per group) every 4 weeks for 2 months resulted in increased muscle section area compared to the vehicle-treated group as detected by magnetic resonance imaging (MRI) (Fig. 5A). In addition, the rate of body weight increase relative to baseline was also higher in the GYM-cyFc group than in the vehicle group (Fig. 5B). Rapid and drastic reduction in the level of total plasma myostatin, which reflects the sweeping function of GYM-cyFc, was observed after the 1st and 2nd doses of the antibody (Fig. 5C). No reduction in plasma myostatin levels was observed in the vehicle treatment group. All doses of GYM-cyFc were well tolerated in the cynomolgus monkeys throughout the duration of the study. Preliminary toxicological investigation revealed no significant adverse pathological effects or abnormalities in hematology and blood chemistry.

## Discussion

Several muscular disorders such as Duchenne muscular dystrophy (DMD), which is characterized by progressive skeletal muscle wasting and weakness leading to death from respiratory and cardiac impairment, still do not have effective therapy^43^. Current therapeutic options for DMD, such as Translarna^44^ and Exondys 51^45^, are limited, as these treatment strategies are designed to treat only a small fraction of patients that carry a particular type of mutation in the dystrophin gene^46^. Furthermore, additional clinical evidence is still required to confirm their clinical benefits. The number of therapeutic agents for muscular atrophy is also limited. In addition to muscular atrophy caused by muscle disuse due to hospitalization after surgery^47^, sarcopenia or age-related muscular atrophy^48^ is considered a growing medical risk in the aging global population. Myostatin has long been considered an attractive target for the treatment of these muscular diseases due to the remarkable phenotypes caused by genetic defects in myostatin, such as muscular hypertrophy and gain of strength^1,4–8,49^. Accordingly, several anti-myostatin inhibitors have been developed and tested in clinical studies. These include a series of antibodies such as MYO-029/stamulumab^50^, PF-06252616/domagrozumab^19^, and LY2495655/landogrozumab^16,17^, and engineered adnectin, BMS-986089 (Clinicaltrials.gov_NCT02515669). Therapy using LY2495655 and bimagrumab led to increased appendicular lean mass in patients who had undergone total hip arthroplasty^17^ or total lean body mass in those with sporadic inclusion body myositis (sIBM) (ClinicalTrials.gov_NCT01925209). However, both antibodies failed to improve physical performance (in the Timed Up and GO, stair climb power, and 6-min walk tests), which is relevant for enhancing the patients’ quality of life. Therefore, new therapeutic approaches that are more effective in improving patient physical function are still needed.

Myostatin gene knockouts confer remarkable muscle strength enhancement in animals, including the DMD mouse model^51^. However, current anti-myostatin agents do not have the same effects as myostatin gene knockouts, suggesting that these agents are not efficiently targeting myostatin and are therefore not maximizing the effects of myostatin-targeted therapy. We hypothesized that the cross-reactivity of these agents to GDF11 contributes to their insufficient activity. Myostatin and GDF11 belong to the activin/inhibin subclass of the TGF-β superfamily and utilize the same set of receptors^31,52^. This suggests that both ligands have overlapping physiological roles, but there are clear differences, such as in expression profiles (myostatin is almost exclusively found in skeletal and cardiac muscle, whereas GDF11 is found in a broader range of tissues)^53,54^, and in the phenotypes of gene knockouts (*Gdf11* knockout in mice results in early neonatal death)^55^. Given that the role of GDF11 in muscle growth and strength is unclear^26–30^, we attempted to examine the possible advantages of myostatin-specific blockade. We also hypothesized that incomplete neutralization of myostatin function in muscle tissues, probably due to high levels of myostatin in the muscle microenvironment and poor antibody penetration, is another issue for conventional anti-myostatin agents. We aimed to overcome this problem by generating an antibody with a sweeping function to reduce myostatin levels in the muscle. Based on our hypotheses, we generated GYM329 and surrogate antibodies that specifically inhibit myostatin signaling and possess the sweeping function. We have demonstrated that GYM329 and its surrogates exhibit superior ability to enhance muscle strength compared to three clinically evaluated anti-myostatin agents. These superior effects were seen in three different muscle wasting disease mouse models, including a DMD model, an aged model, and a hindlimb suspension-induced atrophy model, which demonstrates the potency of this antibody in treating muscular diseases.

Combined administration of a GDF11-neutralizing antibody with GYM329 in the muscular atrophy model diminished the superior effects of GYM329. Moreover, administration of recombinant GDF11 in the atrophy model reversed the reduction in muscle strength induced by hindlimb suspension. These findings suggest that the specific inhibition of myostatin, and not of GDF11, is beneficial for the treatment of muscle wasting disorders. Despite sharing receptors, GDF11 and myostatin seem to act in opposite directions in muscle strength enhancement. Recently, it was reported that GDF11 promotes osteogenesis as opposed to myostatin by activating BMP signaling through Smad 1/5/9^56^. It was also shown in endothelial cells that GDF11 activates Smad 1/5/8^57^, whereas myostatin and GDF11 have been previously thought to activate Smad 2/3. BMP signaling has been suggested to act as a positive regulator of skeletal muscle mass thorough Smad1/5-mediated activation of mTOR signaling^58^. Although these findings suggest possible differential signaling mechanisms between myostatin and GDF11, its details are still elusive. Further investigation of the molecular mechanism of GDF11 in the musculature would deepen our understanding of muscle growth and strength regulation.

Recently, another group has reported an antibody with myostatin-specific blockade function enabled through binding of the latent form of myostatin^59^. Although they have shown that their antibody has the capacity to increase muscle mass in vivo using a steroid-mediated atrophy mouse model, they did not compare the effects of their antibody with those of anti-mature myostatin antibodies that have GDF11 cross-reactivity. Our study is therefore the first to demonstrate the advantages of myostatin-specific blockade in treating muscle diseases.

We have also demonstrated that the sweeping function of GYM329 is a significant contributor to its superior efficacy by comparing GYM329 to a non-sweeping counterpart with identical neutralizing potency. Reports state that myostatin exists predominantly in the pro-myostatin form in the muscle^13^. Our immunohistochemical analysis showed that the levels of pro-myostatin and/or the latent form of myostatin are elevated in the muscular atrophy model. Myostatin levels were clearly reduced by treatment with GYM329 but not with the non-sweeping counterpart. FcγRIIb is expressed on endothelial cells and immune cells that are predominantly found in muscle tissues^60,61^; therefore, we hypothesize that the sweeping of myostatin occurs in these cells, although further investigation is needed to confirm this hypothesis. Reduction of myostatin via the sweeping function would enable GYM329 to block myostatin signaling more completely even with limited antibody penetration into the muscles, which could explain why GYM329 exhibited more potent effects. Additional studies on the distribution of GYM329 and the status of myostatin signaling blockade in the muscle microenvironment are necessary to confirm this hypothesis.

Although muscle function analysis was not performed in the study using cynomolgus monkeys due to technical limitations, obvious muscle increment was observed in the monkeys treated with the GYM329 surrogate. In the preliminary analysis of safety in the same experiment, no obvious toxicological effects were observed. Total plasma myostatin was also strongly reduced by the GYM329 surrogate antibody, demonstrating that the sweeping function works in different species, as expected.

Notably, the superior effect of GYM329 is more evident in muscle strength improvement than in muscle mass increment. This characteristic is important for the treatment of patients with disorders such as muscular dystrophy or atrophy, because recovery of physical function is considered the most desirable benefit of therapy. Therefore, GYM329 is potentially beneficial to patients with muscle diseases. Taken together, the findings we present here justify a clinical evaluation of GYM329 for improving physical function in patients suffering from muscle dysfunction.

## Materials and methods

### Surface plasmon resonance binding assay

Binding kinetics of the antibodies against human, cynomolgus monkey, or mouse latent myostatin were assessed at pH 7.4 and 6.0 at 25 °C using the Biacore T200 (GE Healthcare Life Sciences, Piscataway, NJ). Antibodies were captured onto the Biacore sensor chip CM5 (GE Healthcare Life Sciences) and immobilized with protein L (BioVision). Recombinant human, monkey, or mouse latent myostatin was prepared by two-fold serial dilutions (2 nmol/L to 32 nmol/L). The sensor surface was regenerated using Glycine 1.5 (10 mmol/L glycine–HCl, pH 1.5, GE Healthcare Life Sciences). Kinetic parameters at pH 7.4 were determined by fitting the sensorgrams to the 1:1 binding model using the Biacore T200 Evaluation Software, version 2.0 (GE Healthcare Life Sciences). The pH-dependent binding ability of the antibodies to latent myostatin was evaluated by comparing the dissociation phases of the sensorgrams at pH 7.4 and at pH 6.0. Further details are described in the Supplementary Materials.

### Inhibitory activity assays on myostatin and GDF11 signaling

A reporter gene assay was used to assess the biological activity of active myostatin or GDF11 in vitro. The detection of bioactive myostatin was achieved by monitoring the activation of activin type 1 and 2 receptors in HEK-Blue TGF-β cells (InvivoGen, San Diego, CA), which stably express Smad3/4-binding elements (SBE)-inducible SEAP as the reporter gene. The quantity of SEAP was measured using QUANTI-Blue (InvivoGen). HEK-Blue TGF-β cells were maintained according to the manufacturer’s instructions. The culture medium was changed to the assay medium (DMEM with 0.1% bovine serum albumin (BSA), 100 U/mL streptomycin and penicillin, and 100 μg/mL Normocin), and cells were seeded into 96-well plates before assays. For the activation of latent myostatin, 3 nmol/L human, cynomolgus monkey, or mouse latent myostatin was incubated with 250 ng/mL recombinant human BMP1 (R&D Systems, Minneapolis, MN) and various concentrations of anti-latent myostatin antibody at 37 °C overnight. The sample mixtures were then added to the cells. After incubation for 24 h, cell supernatants were collected and mixed with QUANTI-Blue, and optical density at 620 nm was measured using a colorimetric plate reader. For other assays using active ligands, 5 ng/mL of mature myostatin or mature GDF11 was added to cells with various concentrations of antibodies for 24 h.

### Animal studies

This study was conducted according ARRIVE guidelines (https://arriveguidelines.org/). All procedures associated with this study were reviewed and approved by the Institutional Animal Care and Use Committee (IACUC) in Chugai Pharmaceutical Co., Ltd. The test facility is accredited by the Association for Assessment and Accreditation of Laboratory Animal Care International (AAALAC). Animal care and experiments were performed according to the animal husbandry policy of Chugai Pharmaceutical Co., Ltd. Animals were housed in a temperature- and humidity-controlled room with food and water ad libitum. Whole-body muscle mass was measured using a body composition analyzer based on Time Domain Nuclear Magnetic Resonance (TD-NMR, Minispec LF50H, Bruker Biospin). Whole-body muscle mass was calculated by multiplying muscle mass (%) by bodyweight and dividing by a hundred. Grip strength tests were performed using a digital force meter (GPM-100B, MELQUEST).

### Mouse in vivo studies

The 9-week-old male CB17/ Icr-Prkdc^scid^/CrlCrlj scid/scid mice (SCID, Charles River Laboratories Japan, Kohoku-ku, Yokohama City) were individually subjected to hindlimb suspension according to a previously reported method with slight modifications^62^. Before administration of therapeutic agents on day 0, whole-body muscle mass of all animals was measured by TD-NMR, and appendicular and hindlimb grip strength were measured by a grip strength meter. Based on these values, the animals were selected and allocated to each dosing group so that there was no variation in each group (6 animals per group). After group allocation, each dosing solution was administered at 10 mL/kg of bodyweight (antibody solutions: intravenous, i.v.; GDF11 and myostatin solutions: intraperitoneal, i.p.). Vehicle (150 mmol/L NaCl, 20 mmol/L His-HCl, pH 6.0) was also administrated intravenously to the 6 mice in the control group. The antibody injection studies in the hindlimb suspension had a duration of 1 or 2 weeks as indicated in the figure legends, and the GDF11 and myostatin injection study lasted for 3 days. Control animals were not suspended. At the end of each experiment, the quadriceps, gastrocnemius, soleus, tibialis anterior, and extensor digitorum longus from both limbs were isolated and weighed. The GDF11 study was performed twice independently, with the same study design. Five-week-old male C57BL/10-*mdx* Jic (*mdx*, CLEA Japan, Tokyo, Japan) mice (6 animals per group), and seventy-nine-week-old male C57BL/6J (aged mice, Charles River Laboratories Japan) mice were used (10 animals per group) for the DMD model and the aged mouse study, respectively. The observation duration was 4 weeks after antibody injection in both studies. Change in whole-body muscle mass or grip strength was determined by subtracting the value on the last day from it on day 0.

The concentration of total myostatin and antibodies were measured by electrochemiluminescence (ECL) immunoassay, and each concentration was calculated based on the calibration curve using the analytical software SoftMax Pro (Molecular Devices, San Jose, CA). To measure total myostatin, acidic solution (0.2 mol/L glycine–HCl, pH 2.5) was added to diluted mouse plasma samples to dissociate mature myostatin from bound proteins (such as propeptide and follistatin). The samples were applied into MULTI-ARRAY 96-well plates and incubated with immobilized anti-mature myostatin antibodies. Next, biotinylated anti-mature myostatin antibodies were added and incubated. After incubation with sulfo-tagged streptavidin, the signal was detected using MESO SECTOR S600 (Meso Scale Discovery, Rockville, MD). For measurement of antibody concentration in plasma, diluted plasma samples were applied onto plates (MULTI-ARRAY 96-well) with immobilized anti-human IgG (I9885, Sigma-Aldrich, St. Louis, MO) and incubated. Next, biotinylated anti-human IgG (2040-08, Southern Biotechnology Associates, Birmingham, AL) was added and incubated for 1 h at room temperature. After incubation with sulfo-tagged streptavidin, the signal was detected using MESO SECTOR S600.

### Cynomolgus monkey in vivo studies

The cynomolgus macaques were housed at Shin Nippon Biomedical Laboratories, LTD. (SNBL), Japan. This study was approved by the IACUC and was performed in accordance with the animal welfare regulations at SNBL, which is accredited by AAALAC International. Three-year-old female cynomolgus monkeys (36 animals in total) were used. GYM-cyFc at doses of 1.25, 2.5, or 5 mg/kg was administered intravenously every 4 weeks for 2 months (a total of 3 times per dose level) to 10 female monkeys per group. Vehicle (150 mmol/L NaCl, 20 mmol/L His-HCl, pH 6.0) was also administrated intravenously to 6 female monkeys as the control group. The effects of GYM-cyFc on muscle mass in three muscle groups (*quadriceps femoris, brachialis*, and *erector spinae*) were investigated via MRI (MAGNETOM Allegra, 3T, SIEMENS, Erlangen, Germany) at day 0 (baseline) and at weeks 4 and 8. Blood samples were drawn from the left femoral vein before each dosing. The concentrations of total myostatin and antibodies were measured by the ECL immunoassay as performed in the mouse studies.

### Measurement of muscle myostatin

Quadriceps muscles were obtained from the hindlimb suspension SCID mice. Muscle lysates were prepared using RIPA buffer (Thermo Fisher Scientific, Waltham, MA), and western blot analysis was performed according to a previously reported method^63^ using Human/Mouse/Rat GDF-8/Myostatin Antibody (AF788, R&D systems Inc.) as the primary antibody and GAPDH (D16H11, Cell Signaling Technology) as the internal control. Quantifications were performed with Image-J ver. 1.47J. To detect extracellular latent myostatin secreted locally around the skeletal muscles, whole-mount EDL muscles were obtained from suspended hindlimb SCID mice. EDL muscles were fixed in 4% paraformaldehyde (FUJIFILM Wako Pure Chemical Corporation, Osaka, Japan) for 24 h at 4 °C. Following fixation, muscles were washed in phosphate-buffered saline (PBS) three times (5 min each wash), and incubated with blocking buffer (1% BSA, Sigma-Aldrich; and 1% goat serum, FUJIFILM Wako Pure Chemical Corporation in PBS) for 2 h at room temperature. Anti-latent myostatin antibody (MST1098-rabbit IgG generated in-house and confirmed to not be cross-reactive to GYM329 (data not shown)) was used as the primary antibody. MST1098-rabbit IgG (1 μg/mL) was diluted with blocking buffer, and the samples were incubated with the primary antibody for more than 24 h at 4 °C. After washing with PBS three times (15 min each wash), the secondary antibody, anti-rabbit IgG Alexa Fluor 568 (1:1000, Thermo Fisher Scientific) diluted with blocking buffer, was added, and the samples were incubated for 24 h at 4 °C. The samples were then washed with PBS three times (15 min each wash), and 4′, 6-diamidino-2-phenylindole (DAPI) was used for nuclear staining. Images were obtained using a Nikon A1 confocal microscope (Nikon, Tokyo, Japan). The Z-series images were captured with 1.225 μm step size, and ~ 100 μm thickness. The images were then flattened into a single image for each location using the Nikon software (NIS-elements software). Red areas in individual images were analyzed using Image-J.

### Statistical analysis

JMP 11.2.1 software (SAS Institute, Cary, NC) was used for group allocation prior to administration, with individual exclusions. All statistical analyses were performed with JMP software (Williams’ test, Tukey test, and Student’s *t*-test). Statistical significance was assigned for *P* < 0.05 (Student’s t-test and Tukey test) or *P* < 0.025 (Williams’ test); results are shown as Mean and SEM or SD.

## Data availability

All data associated with this study are present in the main text or the Supplementary Materials. Materials are available from Chugai Pharmaceutical Co., Ltd. under a material transfer agreement.

## Supplementary Information

Supplementary Information.
