# Supplementary material for: Novel myostatin-specific antibody enhances muscle strength in muscle disease models
Source: Sci Rep. 2021 Jan 25;11:2160. doi: 10.1038/s41598-021-81669-8 (PMC7835227; doi:10.1038/s41598-021-81669-8)
Supplement: Supplementary file 1 — Supplementary Information. [file 41598_2021_81669_MOESM1_ESM.docx]

Novel myostatin-specific antibody enhances muscle strength in muscle disease models

Hiroyasu Muramatsu^1^, Taichi Kuramochi^2^, Hitoshi Katada^1^, Atsunori Ueyama^1^, Yoshinao Ruike^1^, Ken Ohmine^1^, Meiri Shida-Kawazoe^1^, Rie Miyano-Nishizawa^1^, Yuichiro Shimizu^1^, Momoko Okuda^2^, Yuji Hori^1^, Madoka Hayashi^1^, Kenta Haraya^1^, Nobuhiro Ban^1^, Tatsuya Nonaka^1^, Masaki Honda^1^, Hidetomo Kitamura^1^, Kunihiro Hattori^1^, Takehisa Kitazawa^1^, Tomoyuki Igawa^2^, Yoshiki Kawabe^1^, Junichi Nezu^1,*^

^1^ Research Division, Chugai Pharmaceutical Co., Ltd., Tokyo 103-8324, Japan.

^2^ Chugai Pharmabody Research Pte. Ltd., 3 Biopolis Drive, #07-11 to 16, Synapse, 138623, Singapore.

*To whom correspondence should be addressed: Junichi Nezu; nezujyn@chugai-pharm.co.jp.

**Supplementary Information** **For**

**Novel myostatin-specific antibody enhances muscle strength in muscle disease models**

Materials and Methods

Fig. S1. Comparison of the effects of GYM-mFc and GYM329 on myostatin activation.

Fig. S2. Sarcopenic characteristics of aged mice.

Fig. S3. Inhibitory effects of landogrozumab and domagrozumab on mature myostatin and mature GDF11.

Fig. S4. Binding properties of the anti-GDF11 antibody.

Fig. S5. Comparison of the recombinant GDF8 and GDF11 in the reporter gene assay.

Fig. S6. Pharmacokinetic study of GYM329 and hMST1032-hIgG1 in mice.

Fig. S7. Uncropped western blot image.

Fig. S8. The sweeping function of GYM329 and GYM-mFc in mice.

Table S1. Binding properties of GYM329 to latent myostatin.

Table S2. Binding properties of GYM329 to human FcγRs.

Table S3. Binding properties of GYM329 to FcRn.

Table S4. Inhibitory activities of GYM329.

Table S5. Binding properties of GYM329 and GYM-cyFc to cynomolgus FcγRs.

**Materials and Methods**

*Expression and purification of recombinant latent myostatin*

Recombinant latent myostatin (NCBI GenBank accession number: NP_005250.1 for human; NP_034964.1 for mouse; and NP_001274552.1 for cynomolgus monkey) was expressed transiently using FreeStyle293-F cell line (Thermo Fisher Scientific). Conditioned medium containing latent myostatin was acidified to pH 6.8, followed by application to a Q Sepharose FF anion exchange column (GE Healthcare Life Sciences). The flow-through was adjusted to pH 5.0 and applied to an SP Sepharose HP cation exchange column (GE Healthcare Life Sciences), and then eluted with an NaCl gradient. Fractions containing the latent myostatin were collected and subsequently applied into a Superdex 200 gel filtration column (GE Healthcare Life Sciences) equilibrated with 1× PBS. Fractions containing the latent myostatin were pooled and stored at −80 °C.

Mature myostatin was purified from the purified latent form. The latent form was acidified by the addition of 0.1% trifluoroacetic acid (TFA) and was applied to a Vydac 214TP C4 reverse phase column (Hichrom Limited, Reading, UK) and eluted with a TFA/CH_3_CN gradient. Fractions containing mature myostatin were pooled, dried, and stored at −80 °C. To reconstitute, mature myostatin was dissolved in 4 mmol/L HCl. Expression and purification of latent and mature myostatin from cynomolgus monkeys and mice, and latent and mature GDF11 were performed in exactly the same way as the human latent myostatin. For *in vitro* binding and inhibitory assays, these purified myostatin and GDF11 were diluted with the appropriate buffer or medium. For *in vivo* studies using GDF11 and myostatin, mature GDF11 (in-house generation) and mature myostatin (PeproTech, Rocky Hill, NJ) dissolved in 4 mmol/L HCl were diluted with 0.1% BSA/PBS for injection.

*Expression and purification of recombinant antibodies*

GYM329 and GYM-cyFc were stably expressed using Chinese hamster ovary cells. Other recombinant antibodies were expressed transiently using FreeStyle293-F cell line (Thermo Fisher). Purification of antibodies from the conditioned medium was performed with the conventional method for purification with protein A. Gel filtration was further conducted if necessary. The sequence information of the anti-mature myostatin antibodies, landogrozumab and domagrozumab, and the anti-ActRII antibody, bimagrumab, were obtained from the International Immunogenetics Information System (IMGT/mAb-DB, <http://www.imgt.org>).

*Antibody generation*

Twelve to sixteen-week-old NZW rabbits were immunized intradermally with mouse latent myostatin and/or human latent myostatin (50–100 μg/dose/rabbit). This dose was repeated 4–5 times over a 2-month period. One week after the final immunization, the spleen and blood samples were collected. Antigen-specific B-cells stained with labelled antigen were sorted using a cell sorter (FACSAria III, BD Biosciences, Franklin Lakes, NJ). They were seeded into plates together with mitomycin C-treated EL4 cells (European Collection of Cell Cultures) and rabbit T-cell-conditioned medium and cultured together for 7–12 days. After cultivation, B-cell culture supernatants were collected for further analysis, and pellets were cryopreserved. The rabbit T-cell conditioned medium was prepared by culturing rabbit thymocytes in RPMI-1640 containing phytohemagglutinin-M (Roche Diagnostics, Basel, Switzerland), phorbol 12-myristate 13-acetate (Sigma-Aldrich), and 2% fetal bovine serum (FBS).

*Antibody screening*

Enzyme-linked immunosorbent assay (ELISA) was used to test the specificity of antibodies in B-cell culture supernatants. Streptavidin (GenScript, Piscataway, NJ) was coated onto 384-well MaxiSorp (Nunc, Thermo Fisher Scientific) plates at 50 nmol/L in PBS for 1 h at room temperature. Plates were then blocked with diluted (5-fold) Blocking One (Nacalai Tesque, Kyoto, Japan). Human or mouse latent myostatin, pre-labeled with NHS-PEG4-Biotin (Thermo Fisher Scientific), was then added to the ELISA plates and incubated for 1 h. The plates were then washed, and the B-cell culture supernatants were added and incubated for another hour. The plates were washed again, and binding was detected by adding goat anti-rabbit IgG-Horseradish peroxidase (A120-111P, Bethyl Laboratories, Montgomery, TX) and 2,2'-azino-bis(3-ethylbenzothiazoline-6-sulfonic acid) (ABTS, SeraCare Life Sciences, Milford, MA). The anti-mature GDF11 antibody was also obtained through the screening of B-cell culture supernatants after immunizing rabbits with mouse mature GDF11 and was generated using the method described above.

*Surface plasmon resonance binding assay*

The kinetic parameters of anti-latent myostatin antibodies against human, cynomolgus monkey Fc gamma receptors were assessed at 20 °C at pH 7.4 using the Biacore T200. Trastuzumab was used as a reference antibody bearing the human IgG1 constant region. Protein L was immobilized onto all flow cells of a CM5 chip using an amine coupling kit (GE Healthcare Life Sciences). Anti-latent myostatin antibodies and analytes were prepared in PB-P+ (phosphate buffer 0.05 mol/L, NaCl 0.15 mol/L, 0.05 w/v% P-20) at pH 7.4. GYM329 or its surrogate antibody was captured onto the sensor surface by protein L. Based on these results, 5.0 μg/mL of antibodies were captured into the immobilized protein L. Antibodies were adjusted to concentrations corresponding to 500 RU for assessing binding with hFcγRIa and cyFcγRIa, and 1000 RU for the other FcγRs. The concentration of each FcγR was adjusted with PB-P+ at pH 7.4 as follows: 0.008 μmol/L for huFcγRIa and cyFcγRIa; and 1 μmol/L for the other FcγRs. The sensor surface was regenerated with glycine (10 mmol/L glycine-HCl, pH 1.5). Biacore T200 Evaluation Software Version 3.0 was used to evaluate the amount of FcγR bound to 1 RU of antibody.

The kinetic parameters of anti-latent myostatin antibodies against human, cynomolgus monkey FcRn were assessed at 25 °C under pH 6.0 using Biacore T200. Trastuzumab was similarly used as a reference antibody. Protein L was immobilized onto all flow cells of a CM5 chip using an amine coupling kit. Anti-latent myostatin antibodies and analytes were prepared in PB-P+ buffer at pH 6.0. GYM329 or its surrogate antibody was captured onto the sensor surface by protein L. Human and cynomolgus monkey FcRn were prepared by two-fold serial dilutions (15 nmol/L to 240 nmol/L) for the GYM329. Human and cynomolgus monkey FcRn were prepared by two-fold serial dilutions (250 nmol/L to 4000 nmol/L) for trastuzumab. The sensor surface was regenerated with glycine buffer. Kinetic parameters were determined by processing and fitting the data to a steady state model using Biacore T200 Evaluation software, version 2.0.

*Measurement of antibody concentrations in SCID mouse plasma*

The concentrations of GYM329 and hMST1032-hIgG1 in mouse plasma were measured by ECL immunoassay. Plates with immobilized anti-human IgG antibody (I9885, Sigma-Aldrich) were prepared by dispensing anti-human IgG antibody onto a MULTI-ARRAY 96-well plate followed by overnight incubation at 4 °C. Mouse plasma samples diluted 100-fold or higher were prepared. Subsequently, they were added onto the plate with immobilized anti-human IgG and allowed to bind for 1 h at room temperature before washing. Next, biotinylated anti-human IgG antibody (2040-08, Southern Biotechnology Associates) was added, and the plate was incubated for 1 h at room temperature before washing. Sulfo-tagged streptavidin was then added and incubated for 1 h at room temperature before washing. Read Buffer T (4×) with surfactant diluted 2-folds with water was immediately added, and the signal was detected using the MESO SECTOR S600. The total antibody concentration was calculated based on the calibration curve using the analytical software SoftMax Pro.


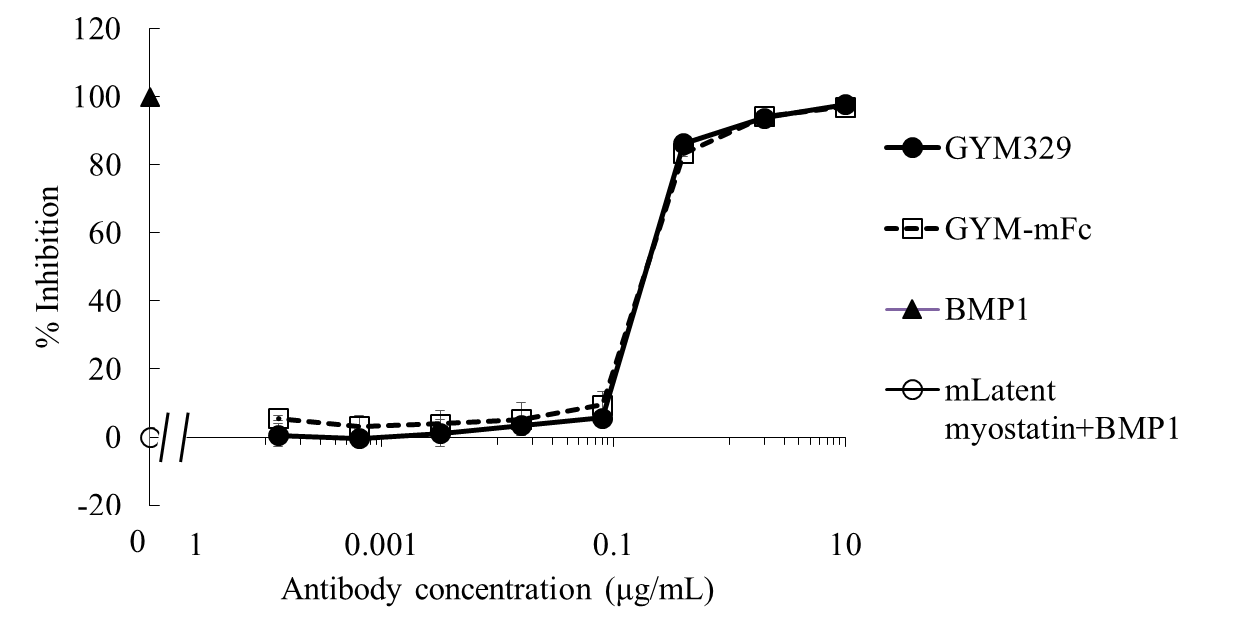


**Fig. S1.** Comparison of the effects of GYM-mFc and GYM329 on myostatin activation. GYM-mFc has the same inhibitory activity against BMP1-mediated activation of latent myostatin as GYM329. Each point represents mean ± SD (n = 3).

**(A)**

**
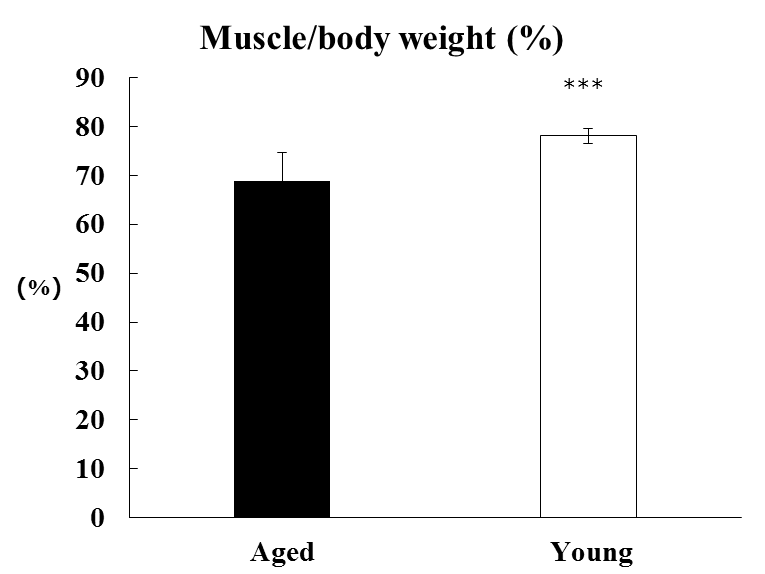
**

**(B)**


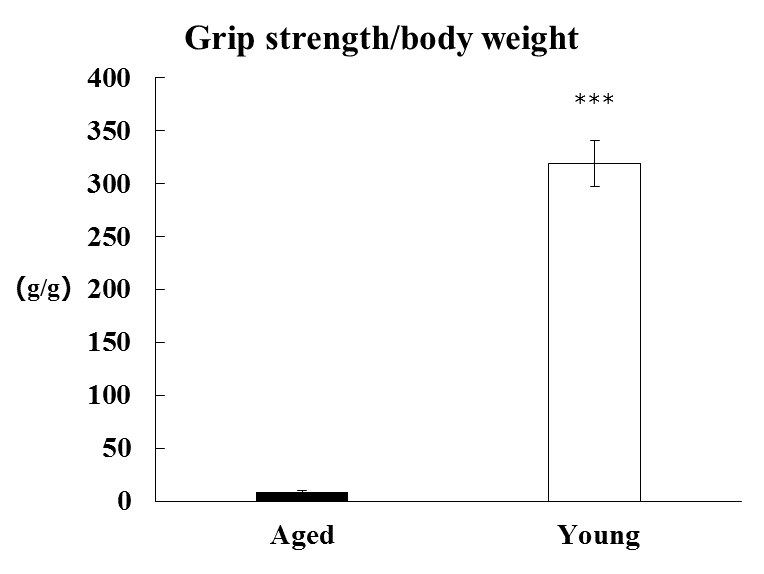


**(C)**

**
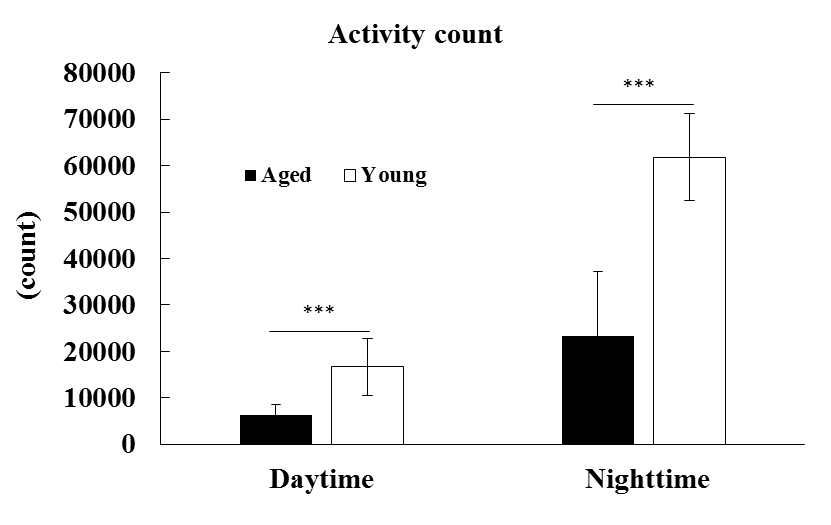
**

**Fig. S2.** Sarcopenic characteristics of aged mice. (**A**) Percentage of muscle mass relative to body weight in aged and young C57BL/6 mice. Data represent mean ± SD; 20-month-old mice: n = 96; 11-week-old mice: n = 6. (**B**) Grip strength per bodyweight in aged and young C57BL/6 mice. Data represent mean ± SD; 20-month-old mice: n = 96; 11-week-old mice: n = 6. (**C**) Mouse activity during daytime and nighttime in aged and young C57BL/6 mice. Data represent mean ± SD; 9-week-old mice: n = 6; 21-month-old mice: n = 9. ^***^*P* < 0.001, with Student’s *t*-test between aged and young C57BL/6 mice.

(**A**)


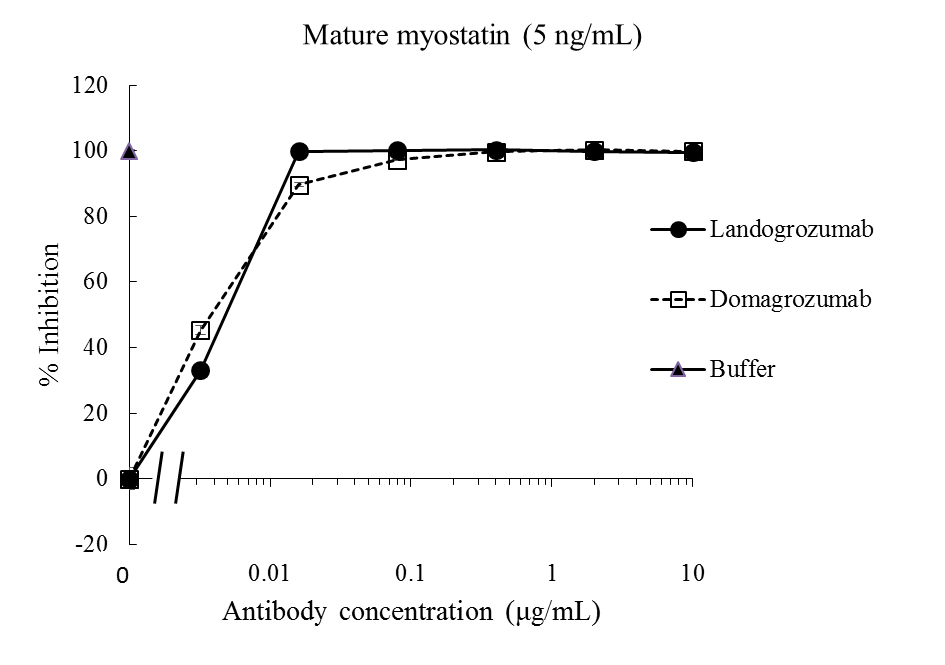


(**B**)


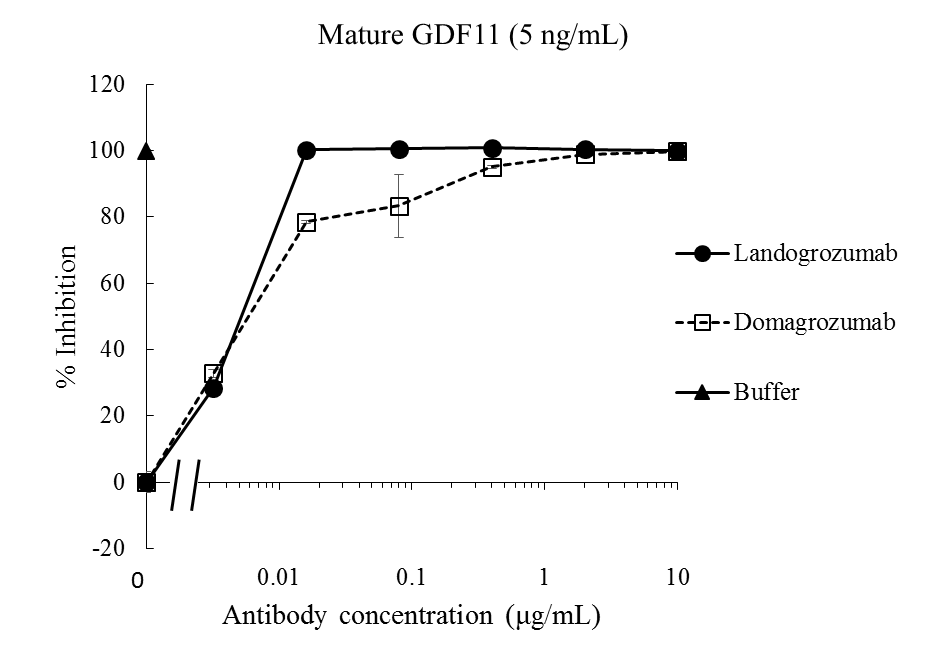


**Fig. S3.** Inhibitory effects of landogrozumab and domagrozumab on (**A**), mature myostatin (5 ng/mL) and (**B**) mature GDF11 (5 ng/mL) (mean ± SD, n = 3).


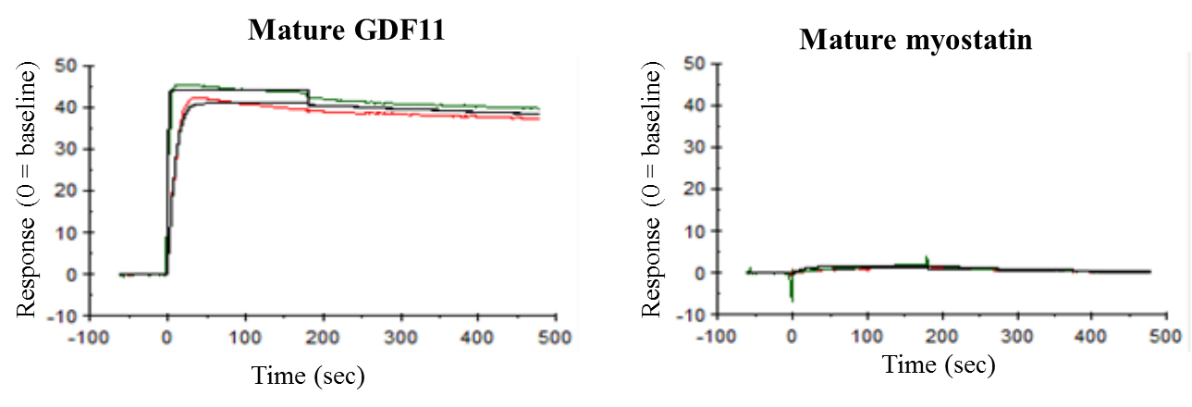


**Fig. S4.** Binding properties of the anti-GDF11 antibody. SPR analysis data showing the binding of the anti-GDF11 antibody to mature GDF11 and to mature myostatin at pH 7.4, 37 °C.


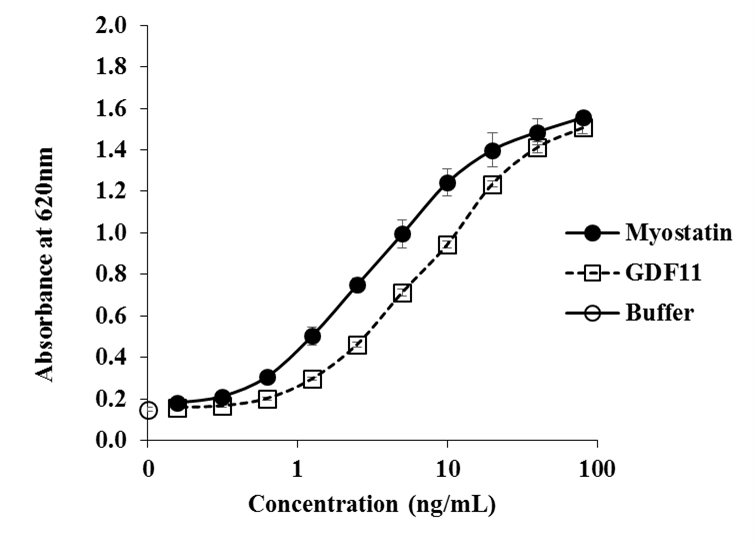


**Fig. S5.** The activities of the recombinant mature myostatin and mature GDF11 in the reporter gene assay. Each point represents mean ± SD (n = 3).


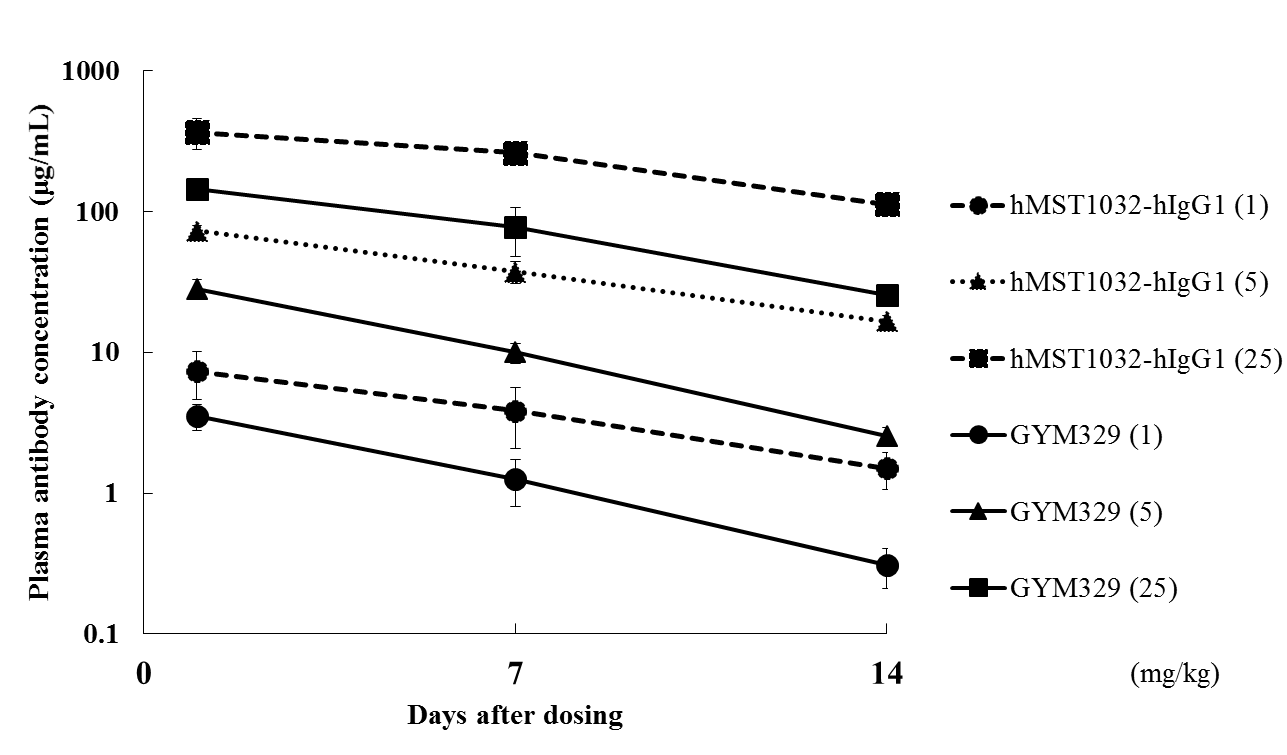


**Fig. S6.** Pharmacokinetic study of GYM329 and hMST1032-hIgG1 in mice. The hMST1032-hIgG1 (non-sweeping reference) and GYM329 were intravenously injected into normal mice (1, 5, or 25 mg/kg bodyweight). Blood was collected from the tail vein on days 1, 7, and 14 after dosing, and the plasma concentration of each antibody was measured by ECL immunoassay. Data represent mean ± SD, n = 6/group.

**
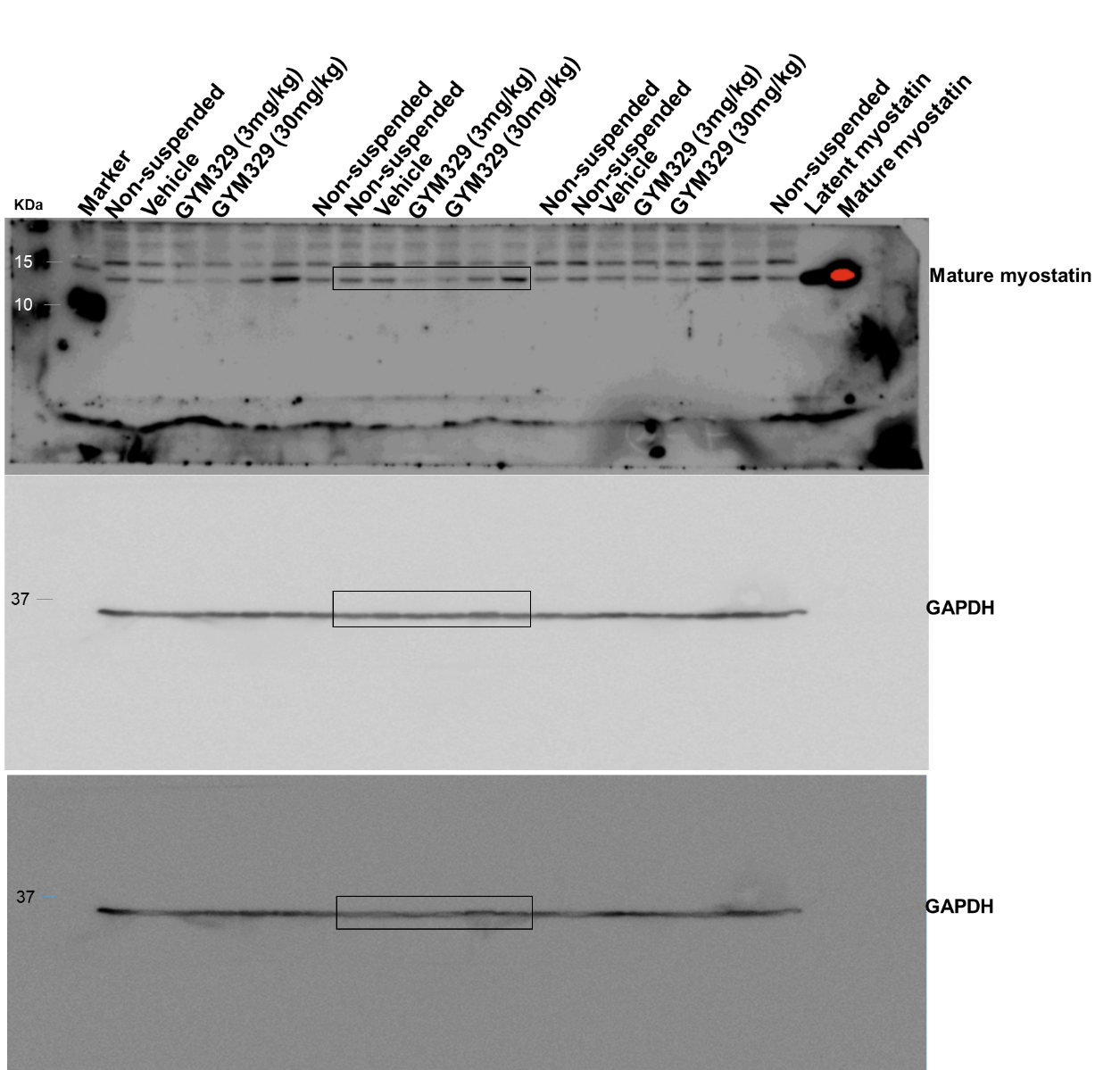
**

**Fig. S7.** Uncropped western blot image. Original images of western blot used to assemble Fig. 4C. Black boxes indicate cropped lanes for mature myostatin and GAPDH. The bottom row shows the membrane of GAPDH for a longer exposure using the same membrane. The figure was compiled using Microsoft PowerPoint (version 15.0.0, Microsoft, Redmond, WA).

**(A)**

**
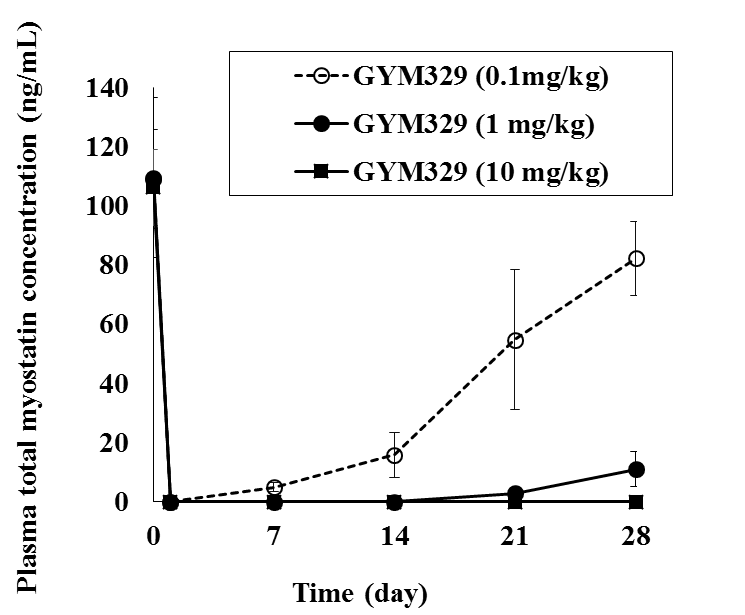
**

**(B)**

**
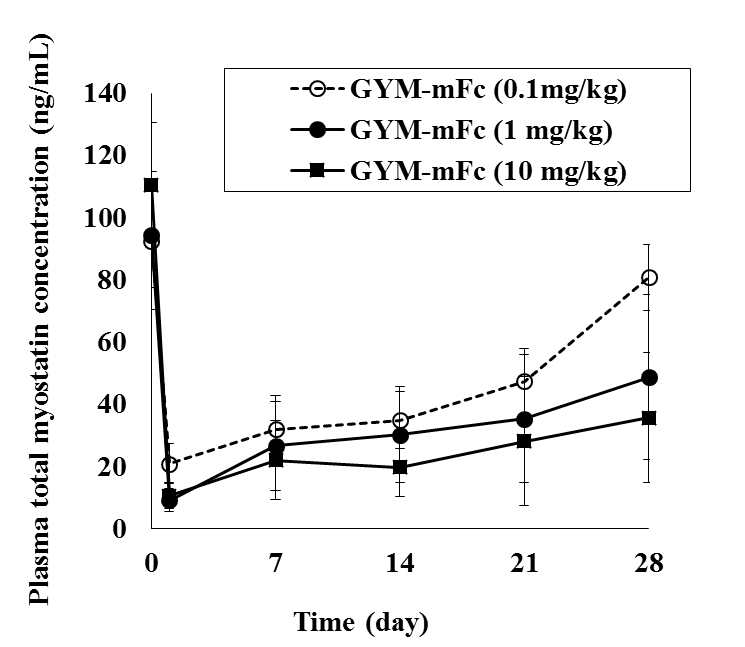
**

**Fig. S8.** The sweeping function of GYM329 and GYM-mFc in normal mice. (**A**) GYM329 and (**B**) GYM-mFc were intravenously administered in normal mice on day 0. Total plasma myostatin concentration was measured by the ECL immunoassay. Data represent mean ± SD (n = 6/group).

**Tables**

| **Latent myostatin** | *k*_a_ (*SD*)   × 10^5^ L/(mol•s) | *k*_d_ (*SD*)   × 10^− 5^ 1/s | *K*_D_ (*SD*)   × 10^− 10^ mol/L |
| --- | --- | --- | --- |
| **Human** | 5.72 (*2.03*) | 8.17 (*2.71*) | 1.44 (*0.05)* |
| **Cynomolgus monkey** | 4.73 (*0.20*) | 6.89 (*0.27*) | 1.46 (*0.06*) |
| **Mouse** | 5.62 (*0.35*) | 7.55 (*0.88*) | 1.35 (0*.24*) |

**Table S1.** Binding properties of GYM329 to latent myostatin. Mean *k*_a_, *k*_d_ and *K*_D_ values of GYM329 at pH 7.4 to latent myostatin. The data represent the means (SD, n = 3).　*k*_a_ = association rate constant; *k*_d_ = dissociation rate constant; *K*_D_ = dissociation constant.

| Human FcγR | Ia | IIa (H) | IIa (R) | IIb | IIIa (F) | IIIa (V) | IIIb NA1 | IIIb NA2 |
| --- | --- | --- | --- | --- | --- | --- | --- | --- |
| **GYM329/IgG_1_** | 0.04 | 0.06 | 0.11 | 5.44 | 0.02 | 0.01 | 0.06 | 0.05 |

**Table S2.** Binding properties of GYM329 to human FcγRs. The binding ratio of GYM329 to a reference wild type human IgG1 (with trastuzumab Fab) on human FcγRs. Note that the amount of FcγRs bound per RU (Resonance Unit) of antibody was measured and the ratio to the wild type human IgG1 was calculated.

| **FcRn** | **GYM329** | **IgG1** |
| --- | --- | --- |
|  | K_D_ (*SD*)  × 10^-7^ mol/L | K_D_ (*SD*)  × 10^-7^ mol/L |
| **Human** | 1.01 (*0.0685*) | 18.7 (*0.336*) |
| **Cynomolgus monkey** | 1.04 (*0.0678*) | 15.3 (*0.448*) |

**Table S3.** Binding properties of GYM329 to FcRn. *K*_D_ values of GYM329 and the reference IgG1 (trastuzumab) to FcRn (pH 6.0). The data represent the means (SD, n = 3).

| **Latent myostatin** | IC_50_ (μg/mL)  BMP1-mediated | IC_50_ (μg/mL)  Spontaneous |
| --- | --- | --- |
| **Human** | 0.182 | 0.135 |
| **Cynomolgus monkey** | 0.133 | 0.107 |
| **Mouse** | 0.139 | 0.102 |

**Table S4.** Inhibitory activities of GYM329. Inhibitory activity of GYM329 on BMP1-mediated and spontaneous activation of latent myostatin presented as IC_50_ values. The IC_50_ values were calculated from each response curve obtained by the reporter gene assay.

|  | cyFcγRIa | cyFcγRIIa1 | cyFcγRIIa2 | cyFcγRIIa3 | cyFcγRIIb | cyFcγRIIIa (S) | cyFcγRIIIa (R) |
| --- | --- | --- | --- | --- | --- | --- | --- |
| **GYM329** | 0.13 | 0.91 | 0.52 | 1.03 | 1.17 | 0.01 | 0.01 |
| **GYM-cyFc** | 0.06 | 1.8 | 2.09 | 2.39 | 2.41 | 0.1 | 0.08 |

**Table S5.** Binding properties of GYM329 and GYM-cyFc to cynomolgus FcγRs. The ratio of GYM329 and GYM-cyFc to the reference wild type human IgG1 (trastuzumab) in cyFcγRs. Amount of bound FcγRs per RU of antibody were measured, and the ratio to the wild type human IgG1 was calculated. Binding to the following receptor polymorphisms were measured: cyFcγRIIIa S and R at position 42. FcγRs = Fcγ receptors, RU = resonance unit.
